# Research on the method of shiitake mushroom picking robot based on CSO-ASTGCN human action prediction network

**DOI:** 10.3389/fpls.2025.1665352

**Published:** 2025-09-11

**Authors:** Daojin Yao, Zichen Yang, Hanxin Chen, Yan Chen, Xiong Yin, Xiaoming Wang

**Affiliations:** School of Electrical and Automation Engineering, East China Jiaotong University, Nanchang, Jiangxi, China

**Keywords:** shiitake mushroom picking robot, human motion prediction, spatiotemporal graph convolutional network, chaos search optimization, human-robot collaboration

## Abstract

**Introduction:**

Automating shiitake mushroom picking is critical for modern agriculture, yet its biological traits hinder automation via target recognition, path planning, and precision challenges. Traditional manual picking is inefficient, labor-heavy, and unsuitable for large-scale production. In human- robot collaboration, computer vision - based human motion prediction enables efficient picking coordination, yet methods like LSTM and static graph networks struggle with robust spatiotemporal correlation capture and long-term stability in complex agricultural settings.

**Methods:**

To address this, we propose the Chaos-Optimized Adaptive Spatiotemporal Graph Convolutional Network (CSO-ASTGCN). First, it integrates three core modules: the Adaptive Spatial Feature Graph Convolution Module (ASF-GCN) for dynamic joint correlation modeling (e.g., wrist-finger coupling during grasping). Second, the Dynamic Temporal Feature Graph Convolution Module (DT-GCN) captures multi-scale temporal dependencies. Third, Chaos Search Optimization (CSO) globally optimizes hyper parameters to avoid local optima common in traditional optimization methods. Additionally, a flexible control system fuses CSO-ASTGCN motion prediction with GRCNN grasp pose estimation to optimize grasping paths and operational forces.

**Results:**

Experiments show our model reduces the Mean Per - Joint Position Error (MPJPE) by 15.2% on the CMU dataset and 12.7% on the 3DPW dataset compared to methods like STSGCN and Transformers. The human - robot collaborative system boosts picking efficiency by 31% and cuts mushroom damage by 26% relative to manual operations.

**Discussion:**

These results validate CSO - ASTGCN’s superiority in spatiotemporal modeling for fine - grained agricultural motions and its practical value in intelligent edible fungi harvesting.

## 1 Introduction

There are over 14,000 species of mushrooms worldwide, accounting for 10% of all existing species on Earth. Among them, more than 2,000 are safely edible, and approximately 700 are known to possess pharmacological activity (Lin et al., 2024). As an important agricultural product with both economic and nutritional value, shiitake mushrooms have seen mechanized operations realized in edible fungus production under China’s bag-based cultivation mode. However, the harvesting stage, which is the most labor-intensive, still faces a situation of “no available machinery” (Wang M. Y. et al., 2022). Nevertheless, the unique biological characteristics of mushrooms pose significant challenges to automated harvesting: their mimicry traits, where the texture and color of the cap are highly similar to those of their growing environment (such as mushroom sticks and culture media), lead to difficulties in target recognition; the dense growth distribution increases the complexity of gripping path planning; and the physical property of fragile and vulnerable mushroom tissue imposes stringent requirements on the precision and flexibility of robotic operations (Yang et al., 2024). Traditional harvesting, relying on manual labor, is not only inefficient and labor-intensive but also fails to meet the demands of modern agriculture for standardized and large-scale production (Yin et al., 2025). Therefore, the development of intelligent harvesting robots with human-robot collaboration capabilities has become an inevitable trend.

In the human-robot interaction system of mushroom harvesting robots, computer vision-based human motion prediction is the core technical support for achieving efficient human-robot collaboration. By accurately inferring the operator’s movement intentions and trajectories, the robot can adjust gripping strategies in advance, optimize action paths, thereby achieving seamless cooperation with human operators, improving harvesting efficiency, and reducing the damage rate of mushrooms. However, human motion prediction still faces multiple technical bottlenecks in complex harvesting scenarios: how to effectively capture the spatiotemporal correlation of posture changes in physical/virtual environments (Sang et al., 2019), and insufficient stability in long-term prediction caused by inadequate modeling of dynamic spatiotemporal correlations. Early studies mostly adopted time-series processing technologies such as autoregressive models (Taylor et al., 2010) and Kalman filters (Lehrmann et al., 2014). These methods rely on linear assumptions and fixed time-series windows, showing good performance in scenarios with periodic and simple movements, but are limited by their ability to model nonlinear dynamic features, making them difficult to adapt to the prediction needs of complex and irregular movement patterns. With the development of deep learning, prediction models based on convolutional neural networks (Li et al., 2018; Liu et al., 2020) and recurrent neural networks (Al-aqel and Khan, 2020; Guo and Choi, 2019; Martinez et al., 2017) have significantly improved prediction accuracy through spatiotemporal feature extraction. However, due to their difficulty in explicitly modeling the complex spatial interaction relationships between human joints, they have limitations in characterizing the dynamic coupling characteristics of joints.

Against this backdrop, Graph Convolutional Networks (GCNs) have provided new insights for human motion prediction. Mao et al. (2019) innovatively encoded temporal information in the trajectory space rather than the traditional posture space, achieving joint modeling of spatiotemporal features of joints by avoiding manually defining the range of temporal dependencies. Cui et al. (2020) proposed a dynamic skeleton graph representation method, which utilized the topological properties of natural connections between joint pairs to realize implicit association modeling of geometrically separated joints. However, existing human motion prediction methods have these issues, especially GCN-based approaches: hyperparameter optimization relies on traditional algorithms like particle swarm optimization and genetic algorithms, which easily fall into local optima (Zhang and Wang, 2019) and lead to suboptimal model performance in non-periodic picking motions; fixed graph structures and static spatiotemporal modeling fail to capture dynamic joint correlations such as the coupling between wrist and finger movements when gripping mushroom stipes, as well as multi-scale temporal dependencies like sudden changes in picking speed due to dense mushroom clusters, resulting in accumulated errors in long-term prediction.

Despite progress in specific scenarios, existing methods still have notable limitations in complex dynamic agricultural picking scenarios. Traditional sequence models such as RNN/LSTM (Martinez et al., 2017) rely on temporal dependency modeling, which struggle with gradient issues when handling nonlinear motions like wrist twisting during picking and fail to explicitly model joint coupling relationships. Among graph neural networks, STSGCN (Sofianos et al., 2021) uses fixed graph structures, unable to adapt to dynamic changes in joint correlations during picking—for instance, adjustments in arm-torso coordination with varying mushroom cluster density. DSGCN (Fu et al., 2023), while improving adaptability by learning joint weights, suffers from increased joint localization errors with prediction steps in scenarios where the operator’s hand is occluded by mushroom clusters, raising robot grasping failure rates. Transformer models (Guo et al., 2023) optimize long-term temporal modeling via self-attention but face high computational complexity, leading to delays in picking scenarios, and lack precision in capturing fine movements like fingertip adjustments.

Existing ASTGCN models have shown effectiveness in general human motion prediction tasks, such as pedestrian trajectory forecasting in smart cities (Sofianos et al., 2021) and motion planning in industrial assembly lines (Dang et al., 2021). However, these scenarios differ fundamentally from agricultural mushroom harvesting: motion patterns in harvesting are more fine-grained and highly coupled with target objects like mushrooms, while the complex background—such as dense mushroom clusters and varying light in greenhouses—exacerbates noise in motion data. Introducing ASTGCN into agricultural harvesting thus requires targeted optimizations: we enhance spatial sensitivity to capture micro-motions of fingers and adjust temporal modeling to adapt to the non-periodic nature of picking actions, addressing the gap between general motion prediction and agricultural-specific demands.

**Figure 1:**
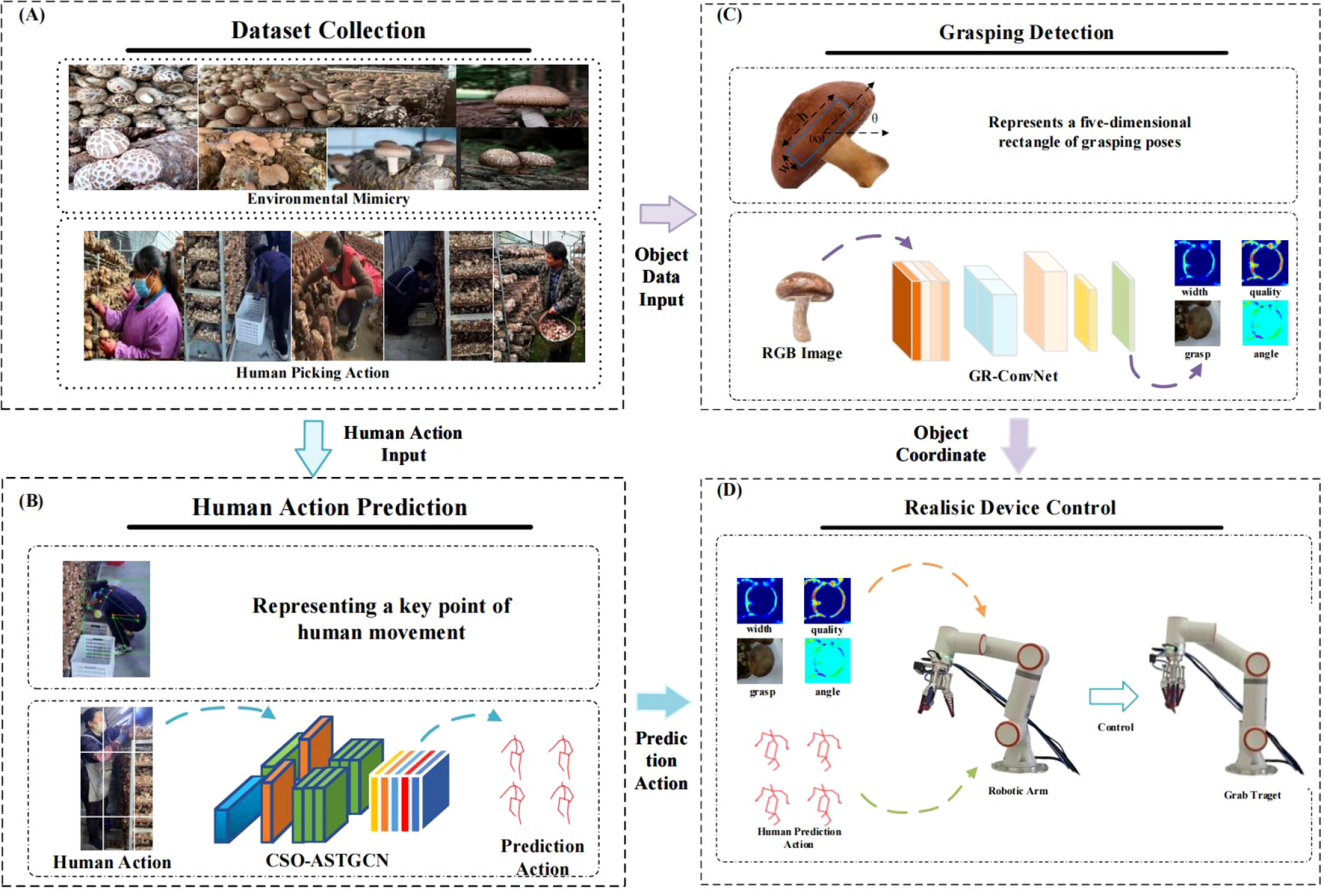
System architecture diagram: **(A)** Collecting human motion and mushroom data, **(B)** Creating a human motion dataset and generating predicted human motions, **(C)** GR-ConvNet algorithm and generating grasping frames, **(D)** Designing the flexible control of the human-robot collaborative shiitake harvesting robot based on the model.

To address existing human motion prediction methods, especially GCN-based approaches, face two critical limitations in complex agricultural scenarios, we propose the a Chaos-Optimized Adaptive Spatiotemporal Graph Convolutional Network (CSO-ASTGCN), which integrates chaos search optimization with adaptive spatiotemporal graph convolution, establishing a synergistic mechanism between hyperparameter optimization and dynamic feature modeling, and applies it to the mushroom harvesting robot system, as shown in **Figure 1**. The specific contributions are as follows:

1. The Chaos-Optimized Adaptive Spatiotemporal Graph Convolutional Network (CSO-ASTGCN) is proposed, which integrates the Adaptive Spatial Feature Graph Convolution Module (ASF-GCN), Dynamic Temporal Feature Graph Convolution Module (DT-GCN), and Chaos Search Optimization (CSO) algorithm. The CSO algorithm is used for global optimization of key hyperparameters such as learning rate and batch size, solving the problem that traditional optimization algorithms are prone to falling into local convergence. By means of ASF-GCN and DT-GCN, the ability to extract spatial features and capture temporal dependencies is enhanced respectively, realizing accurate modeling of the spatiotemporal correlations of human motion and improving the accuracy of human motion prediction in complex harvesting scenarios.
2. A spatially sensitive feature enhancement mechanism is introduced. Through dynamic calculation and feature fusion of channel and spatial sensitivity, the model’s ability to extract dynamic spatial constraints of human joints is strengthened. This mechanism improves the accuracy of capturing key joint features in irregular motions through selective perception of features in horizontal and vertical directions, effectively enhancing the stability of long-term motion prediction.
3. A flexible control system for human-robot collaborative shiitake harvesting robots is designed. It combines the human motion prediction results of CSO-ASTGCN with the grasp pose estimation of GRCNN, and optimizes the robot’s grasping path and operation force by pre-perceiving the operator’s motion intentions. This system realizes synchronous tracking of position and velocity, reduces human-robot interaction delay, minimizes the mushroom damage rate, and improves harvesting efficiency and the fluency of collaborative operations, as shown in **Figure 1D**.

The structure of this paper is arranged as follows: Section 1 elaborates on the design of the CSO-ASTGCN prediction model, including the specific implementation of the Adaptive Spatial Feature Graph Convolution Module (ASF-GCN), Dynamic Temporal Feature Graph Convolution Module (DT-GCN), and Chaos Search Optimization (CSO) algorithm; Section 2 introduces the GR-CNN-based grasp pose estimation method, focusing on the architecture of the GR-CNN grasping network; Section 3 evaluates the performance of the proposed model in human motion prediction and grasp pose estimation tasks through comparative experiments, ablation experiments, and visualization analysis; Section 4 summarizes the research results, points out the existing limitations, and prospects future research directions.

## 2 Related work

### 2.1 Research progress in agricultural robots and human-robot collaboration

Human-robot collaboration technology in agricultural robots has become a core direction to break through the bottlenecks of operations in complex scenarios. Existing studies have shown that traditional agricultural robots mostly rely on preset trajectories or fixed rules., the tracked walking mechanism and four-bar linkage picking device of blueberry picking robots (Wang et al., 2025). Although they can achieve basic automated operations, their adaptability is insufficient in dynamic human-robot interaction scenarios. For instance, in pepper picking, operators need to adjust the manipulator posture in real time to avoid occlusion by branches and leaves, but traditional pre-programmed control struggles to respond to such dynamic demands (Zhou, 2025).

In recent years, the introduction of embodied intelligence technology has provided new ideas for dynamic collaboration. The multimodal fusion perception framework proposed by Wei et al. (2025), which synergistically captures environmental and human actions through visual, force, and auditory sensors, has improved the harvesting efficiency of tomato picking robots by 28.7%. However, the system still shows weakness in long-term sequence modeling of operators’ continuous actions. Fan et al. (2025) integrated RGB-D information with the improved YOLOv8s model in tomato picking, achieving a fruit positioning error of < 4mm, but it does not involve the prediction of human-robot action coupling relationships, making it difficult to support real-time collaborative decision-making.

In terms of human-robot collaboration strategies, existing methods have two limitations: first, they mostly focus on a single operational link (such as recognition or grasping) and lack integration of the entire “perception-decision-execution” chain (Liu et al., 2025); second, they insufficiently consider agricultural scene-specific dynamic interferences (e.g., lighting changes, fruit occlusion), leading to reduced collaboration robustness. For example, in mushroom picking, the fine movements of operators holding tools (such as rotational cutting) and the trajectory planning of the robot end-effector require millisecond-level synchronization, but existing models struggle to capture such high-frequency dynamic correlations (Wei et al., 2025).

### 2.2 Human motion prediction

The evolution of human motion prediction technology has advanced from traditional sequence models to deep learning models, yet significant adaptability issues persist in agricultural human-robot collaboration scenarios.

Traditional sequence models (typified by RNN/LSTM) achieve motion prediction through temporal dependency modeling (Martinez et al., 2017). However, they suffer from the gradient vanishing problem when handling nonlinear motions, such as wrist twisting during mushroom picking—with long-term prediction errors accumulating to over 15% (Ansari et al., 2025). Their serial computing nature causes inference delays exceeding 200ms, failing to meet agricultural robots’ real-time demands (Liu et al., 2025). While gating mechanisms like GRU partially mitigate gradient issues, they still struggle to model complex joint couplings, such as arm-torso coordination.

Graph Neural Networks (GNNs) dominate due to their skeletal topology modeling strengths. For example, Spatio-Temporal Separable Graph Convolutional Network (STSGCN) (Sofianos et al., 2021) uses spatiotemporal separable convolutions but has a fixed graph structure, unable to adapt to dynamic joint correlation changes during picking. Dynamic models like DSGCN (Fu et al., 2023) improve adaptability by learning joint weights, but in densely occluded scenarios, joint localization errors grow exponentially with prediction steps, raising robot grasping failure rates when an operator’s hand is partially occluded by mushroom clusters.

Transformer models optimize long-term temporal modeling via self-attention, reducing MPJPE by 6.7% on datasets like 3DPW (Guo et al., 2023). However, their quadratic complexity leads to 500ms delays in 30fps picking scenarios, more than the 100ms safety threshold (Ansari et al., 2025). They also lack precision in capturing fine local motions, with errors increasing by over 20% in tool-based tasks (Chen, 2025).

Domain adaptation remains a critical flaw: existing models rely on general datasets lacking agricultural-specific actions like tool-assisted picking. Cao Haotian (2025) notes a 35% action distribution gap between general datasets and agricultural scenarios, causing transfer biases. For instance, CMU’s “reaching” is often tool-free and linear, while mushroom picking’s “scissor-cutting” involves complex wrist rotations and fingertip adjustments—details traditional models fail to capture.

## 3 Materials and methods

### 3.1 Design of CSO-ASTGCN prediction model

The proposed ASTGCN model is structured to capture dynamic spatiotemporal features of human motion, with two core modules forming its backbone. The ASF-GC module dynamically updates the spatial constraint matrix (**Equation 16**) by fusing static skeleton priors (D_s_) and real-time joint interactions (M_s_), enabling adaptive modeling of context-dependent spatial correlations—such as wrist-finger coordination during gripping motions. Complementing this, the DT-GC module operates symmetrically in the temporal dimension (**Equation 18**); it replaces fixed time windows with sliding convolutions to capture temporal patterns, including non-periodic variations like pauses in movement. This model is integrated with the CSO algorithm, which optimizes key hyperparameters to modulate the weight updates of ASF-GC and DT-GC modules. The following sections detail the implementation of these components, along with experimental materials and procedures.

**Figure 2:**
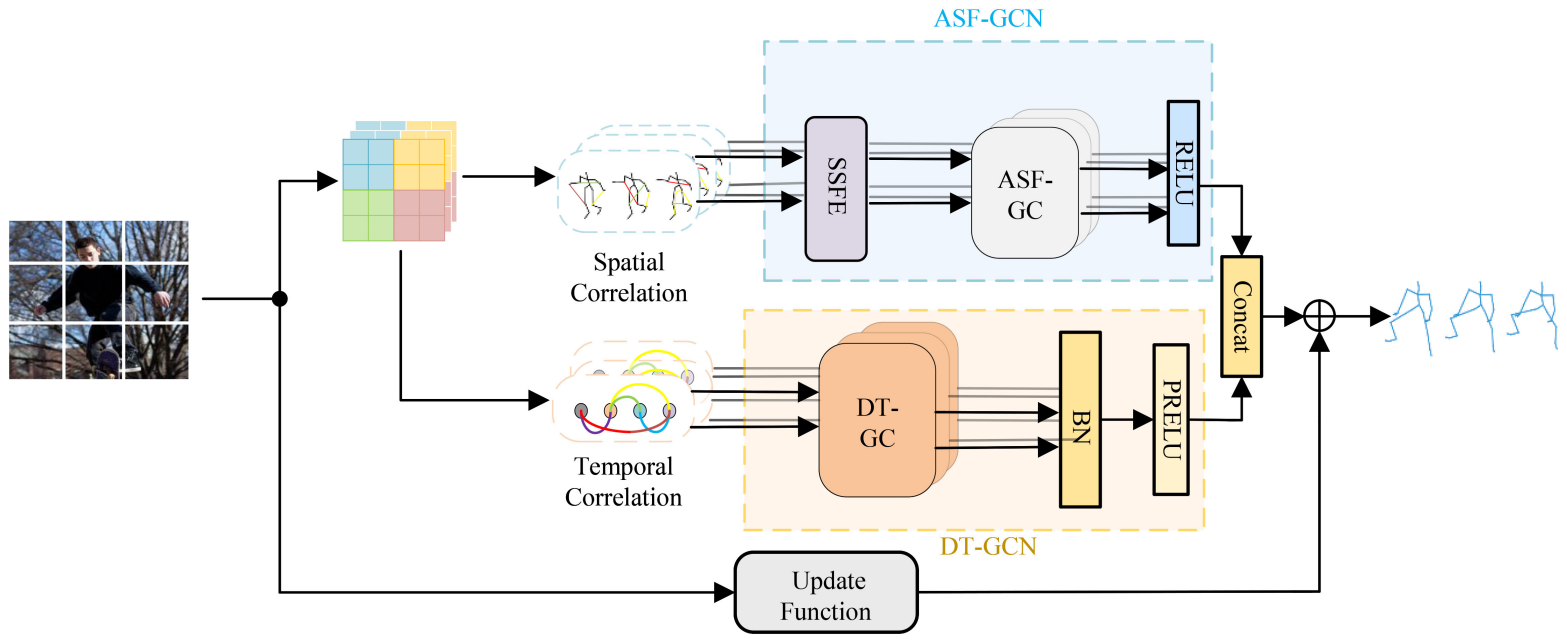
ASTGCN model architecture diagram.

The architecture of the CSO-ASTGCN model proposed in this paper is mainly composed of the Adaptive Spatial Feature Graph Convolutional Module (ASF-GCN), Dynamic Temporal Feature Graph Convolutional Module (DT-GCN), and Chaos Search Optimization (CSO). The CSO algorithm generates initial solutions through chaotic mapping, and after iteratively optimizing hyperparameters, feeds them back to the training process. The framework of the ASTGCN model is shown in **Figure 2**.

Input image features are first converted into three parts: dynamic spatial features, dynamic temporal correlations, and static constraints. The ASF-GCN module encodes the input dynamic spatial features to enhance the capability of spatial feature extraction. The DT-GCN module processes dynamic temporal features, captures temporal dependencies and variation trends, and models dynamic temporal correlations. Static constraints are shared as human motion priors among all samples, and dynamic spatiotemporal graph convolution is introduced to establish dynamic constraint-based temporal modeling and spatial models respectively. A feature enhancement mechanism is incorporated into spatial feature modeling to improve feature extraction capability (Hou et al., 2021), and the predicted results of human motion are outputted.

#### 3.1.1 Chaotic search optimization algorithm

In the CSO-ASTGCN model, the learning rate, batch size, number of network layers, and dropout rate are selected as optimization targets. The learning rate directly regulates the model’s convergence speed and overfitting risk, and its value significantly affects the model’s ability to adapt to scenarios—especially when processing non-periodic dynamic data such as picking actions. The setting of batch size needs to strike a balance between training stability and memory utilization efficiency, and must be adapted to hardware conditions to balance iteration efficiency and the reliability of gradient estimation. The number of network layers determines the depth of feature extraction by the model, and its design needs to achieve a reasonable trade-off between the ability to capture fine-grained joint couplings and computational cost. The dropout rate is used to alleviate overfitting in small-sample scenarios, enhancing the model’s generalization ability by moderately randomly deactivating neurons. The detailed procedure of the Chaos Search Optimization (CSO) algorithm for ASTGCN hyperparameter tuning is presented in **Algorithm 1**.

Algorithm 1 **CSO for ASTGCN hyperparameter tuning.**

**Input:** Search ranges for hyperparameters including learning rate 
$lr\in [lr_{\text{min}},lr_{\text{max}}]$
, batch size 
$bs\in [bs_{\text{min}},bs_{\text{max}}]$
, number of network layers 
$L\in [L_{\text{min}},L_{\text{max}}]$
, dropout rate 
$dr\in [dr_{\text{min}},dr_{\text{max}}]$
, maximum iterations 
$T_{\text{max}}$
, and population size 
N
.
**Output:** Optimal hyperparameter set 
$\theta^{*}=(lr^{*},bs^{*},L^{*},dr^{*})$
.
1: Initialize chaotic sequence via Logistic map (enhance solution diversity)
2: **for** 
i=1~N
 **do**
3: 
$x_{0}(i)∼U(0,1)$
;//Random initial chaotic value
4: **for** 
t=1~50
 **do**//Pre-iterate to stabilize chaotic characteristics
5: 
$x_{t}(i)=4x_{t-1}(i)(1-x_{t-1}(i))$
;//Logistic map formula
6: **end for**
7: **end for**
8: Map chaotic values to hyperparameter space (scale to valid ranges)
9: **for** 
i=1~N
 **do**
10: 
$lr(i)=lr_{\text{min}}+x_{i}(lr_{\text{max}}-lr_{\text{min}})$
;//Scale learning rate
11: 
$bs(i)=\text{round}(bs_{\text{min}}+x_{i}(bs_{\text{max}}-bs_{\text{min}})$
;//Integer hyperparameters
12: 
$dr(i)=dr_{\text{min}}+x_{i}(dr_{\text{max}}-dr_{\text{min}})$
;//Scale dropout rate
13: 
θ(i)=(lr(i),bs(i),L(i),dr(i))
;//Form hyperparameter set
14: **end for**
15: Evaluate initial fitness
16: **for** 
i=1~N
 **do**
17: Train ASTGCN with 
θ(i)
; compute MPJPE;//Validate model performance
18: 
fitness(i)=1/(1+MPJPE)
;//Fitness function definition
19: **end for**
20: 
$\theta^{*}\leftarrow \arg\max(\text{fitness}(i))$
;//Select initial optimal solution
21: 
α←0.5
;//Mutation scaling factor
22: **for** 
$t=1\sim T_{\text{max}}$
 **do**//Main optimization loop
23: Generate mutated solutions
24: **for** 
i=1~N
 **do**
25: 
$x_{i}^{\prime }\leftarrow \text{clamp}(x_{i}+\alpha \times (x^{*}-x_{i})\times \text{rand}(-1,1),0,1)$
;//Bound to [0,1]
26: 
$Mapx_{i}^{\prime }∼\theta^{\prime }(i)$
 as in lines 10 - 12;//New hyperparameter candidate
27: Train ASTGCN with 
θ(i)
; compute 
fitness(i)
;//Evaluate new candidate
28: **if** 
${\text{fitness}}^{\prime }(i)>\text{fitness}(i)then\theta (i)\leftarrow \theta^{\prime }(i);\text{fitness}(i)\leftarrow {\text{fitness}}^{\prime }(i)$
;//Update if better
29: **end if**
30: **end for**
31: 
$\theta^{*}\leftarrow \arg\max(\text{fitness}(i));\alpha \leftarrow \alpha \times 0.95$
;//Shrink search range gradually
32: **end for**
33: **return** *θ*
^*^.

Traditional optimization algorithms (such as particle swarm optimization and genetic algorithm) tend to fall into local convergence, rely on manual parameter tuning, and have low efficiency. For this reason, this paper introduces the Chaos Search Optimization (CSO) algorithm, which utilizes the ergodicity of chaotic mapping to generate high-quality initial solutions and performs global search in the hyperparameter space through a dynamic optimization strategy.

**Figure 3:**
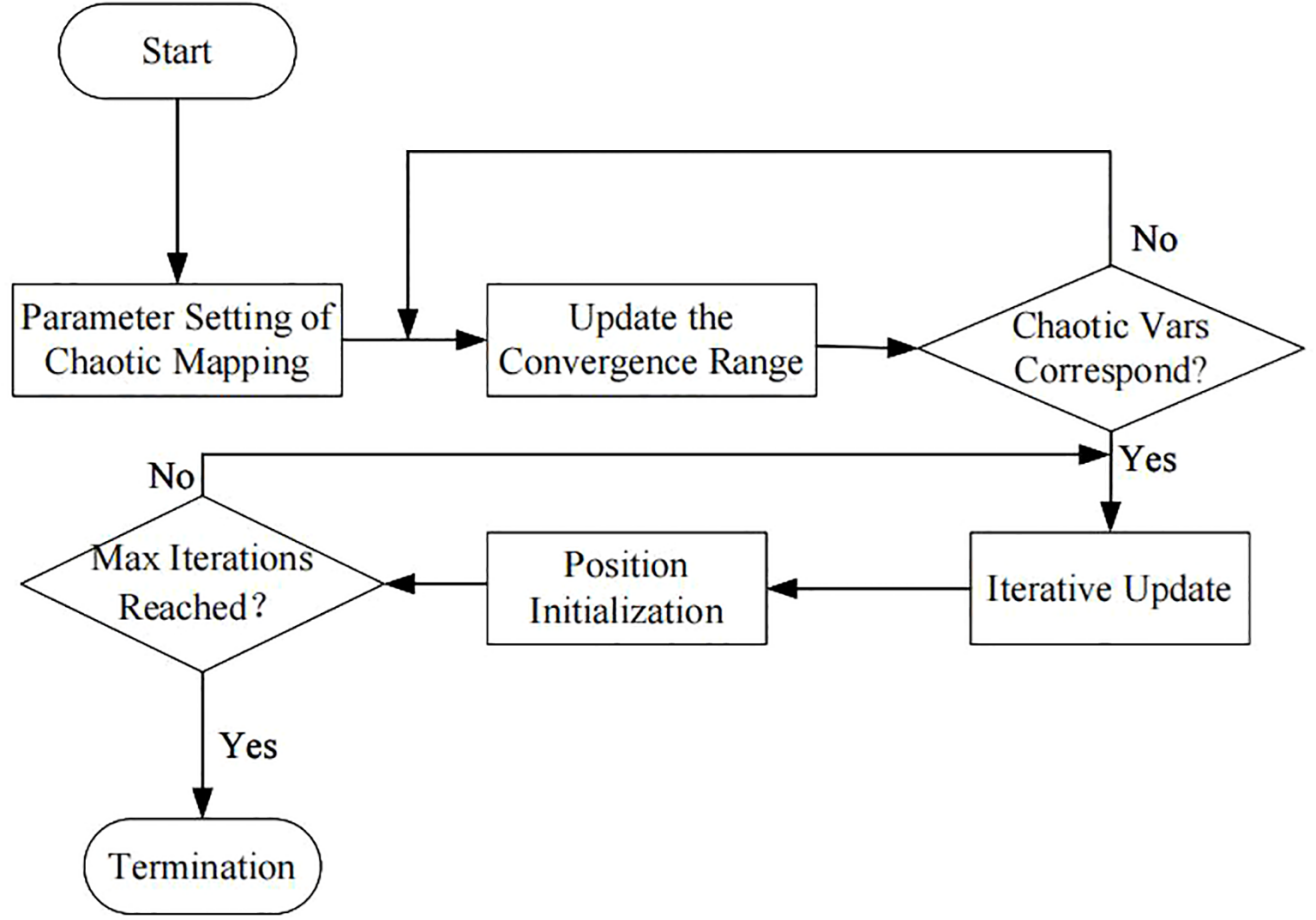
Flowchart of the CSO algorithm.

Chaos Search Optimization (CSO) is an algorithm based on wolf pack optimization. The traditional wolf pack optimization algorithm has poor convergence (Zhang and Wang, 2019), requiring appropriate parameter settings and multiple runs to obtain the optimal solution. By introducing chaotic mapping to generate initial solutions and increasing the diversity of the search, it can automatically acquire optimal parameter settings and reduce the number of algorithm runs, improving the overall operational efficiency of the algorithm. The algorithm flow is shown in **Figure 3**.

CSO first generates diverse solutions via a chaotic mapping mechanism, and the adopted mapping form is as shown in **Equation 1**:


(1) $$x_{n+1}=(a-x_{n}^{2})\cdot \sin(b\cdot \pi \cdot x_{n})$$

Where a and b are parameters controlling chaotic behavior, and $x_{n}$ is the *n*-th chaotic value. After chaotic mapping, a sequence with chaotic characteristics can be generated.

The generated sequences with chaotic characteristics are sorted, and the optimal sequence is selected as the optimal initial solution, i.e., the information of the optimal solution. Additionally, in the *m*-dimensional initial solution space $\theta_{i}=\{\theta_{i1},\theta_{i2},\ldots ,\theta_{im}\}$ , the solution with the optimal objective function value is chosen as the initial optimal solution for each iteration. Then, a portion of the solutions in the solution space (excluding the candidate optimal solution) are treated as matching solutions to seek potentially better solutions within the space. During each iterative matching process, the quality of each solution is determined by evaluating its position. In the convergence process, if the objective function value of a solution exceeds the maximum objective function value again, this solution will replace the current optimal solution for convergence. The distance of each solution in the space is determined as shown in **Equation 2**:


(2) $$d_{\text{n}}=\frac{1}{Rl}\sum_{d=1}^{W}|\underset{d}{\max}-\underset{d}{\min}|$$

Where, l denotes the distance determination factor, *R* represents the number of optimized variables, $\underset{d}{\max}$ and $\underset{d}{\min}$ are the maximum and minimum values of the d-th dimensional variable, respectively.

If all solutions within the solution space are no greater than the maximum objective function value, other matching solutions are then combined to match the potential optimal solution, which is updated as the final optimal solution at the end of each iteration. As each iteration concludes, the convergence range and the quality of solutions are evaluated and updated, and its fitness evaluation function is expressed as shown in **Equation 3**:


(3) $$\text{Euclidean Distance}(x)=\sqrt{\sum_{d=1}^{R}{\left(x_{d}-x_{d}^{*}\right)}^{2}}$$

Where $x_{d}$ is the current solution of the *d*-th dimensional variable, and $x_{d}^{*}$ is the target solution. After the fitness and convergence range are finalized, the three optimal solutions are evaluated, and the optimal output is selected as the final optimal solution of the iteration. Additionally, by setting the chaotic mapping coefficient A, the scaling factor C, and the distance D of the objective function as shown in **Equations 4**–**6**:


(4) $$A=2a\cdot r_{1}-a$$

(5) $$C=2\cdot r_{2}$$

(6) D=|A·ƛ−θ|

Where a and r are both random numbers, $r_{1}$ represents the dynamic range of convergence, $r_{2}$ is used to determine the moving direction from the current solution to the target solution, ƛ denotes the optimal solution, and θ is the convergence parameter for the distance of the objective function. The position of the solution is updated again using chaotic mapping as shown in **Equation 7**:


(7) $$X_{ƛ}=ƛ-B_{ƛ}\cdot D_{ƛ}$$

Where $B_{ƛ}$ is the coefficient for adjusting the convergence step size, and $D_{ƛ}$ is the distance between the current solution and the optimal solution. Finally, when the maximum number of iterations is reached, the optimal solution is evaluated and output as the optimal hyperparameters of the model.

#### 3.1.2 Adaptive dynamic spatio-temporal convolution ASF-GC

##### 3.1.2.1 Spatial sensitive feature enhancement mechanism

**Figure 4:**
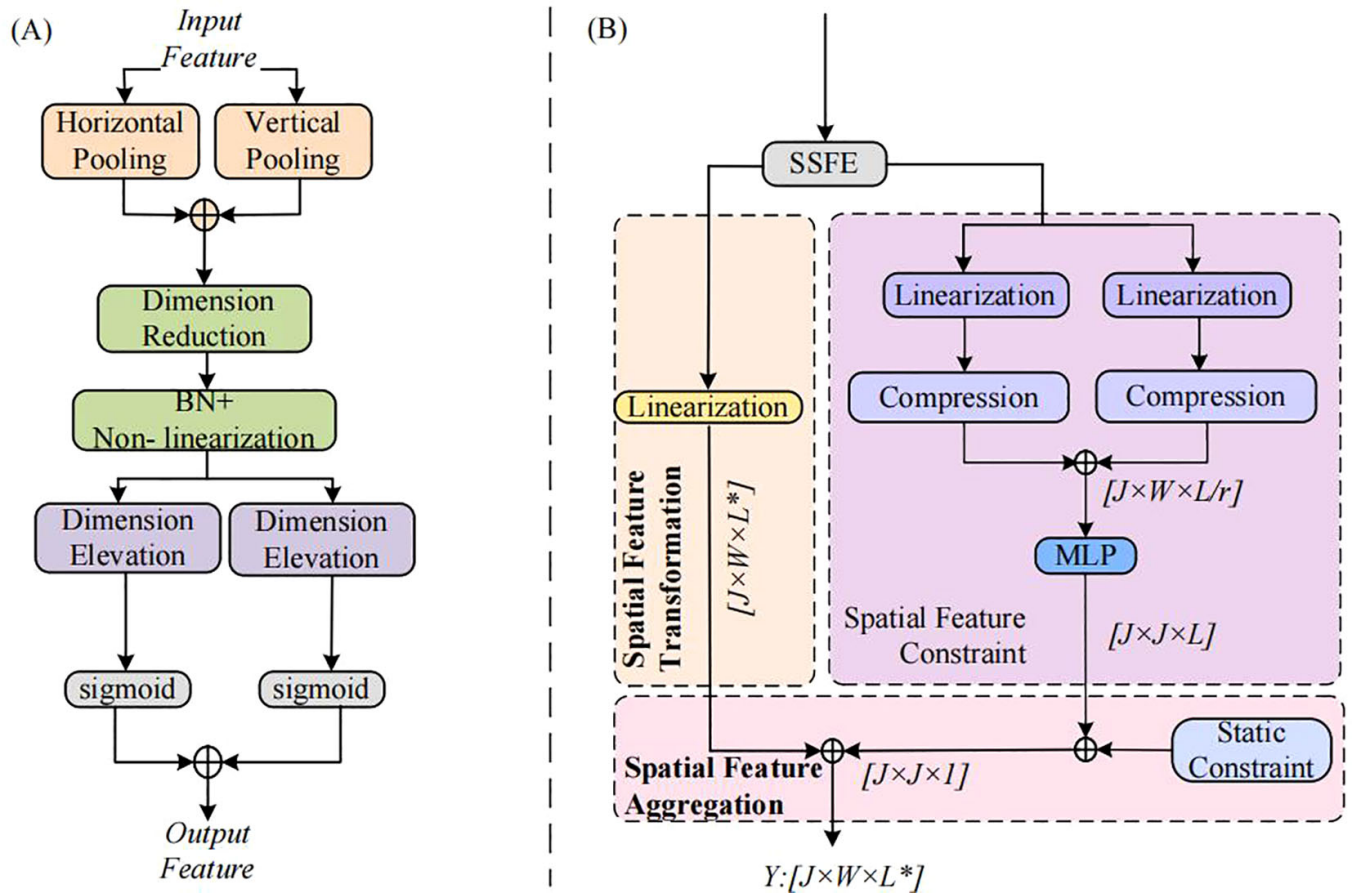
Dynamic Spatial Graph Convolution ASF-GC Module Diagram: **(A)** Spatially-Sensitive Feature Enhancement Mechanism; **(B)** ASF-GC Structure.

In spatial feature modeling, our model automatically focuses on important features related to human motion and filters out irrelevant redundant information by introducing a spatially - sensitive feature enhancement mechanism. Moreover, by weighting different positions of the input features, the model is enabled to pay greater attention to the important constraints of human motioon. Its structural diagram is presented in **Figure 4A**.

The first step of the spatially - sensitive feature enhancement mechanism embeds channel feature information into the input feature tensor $\zeta \in {\mathbb{R}}^{J\times W\times H}$ . For the extracted spatial feature tensor, global pooling is carried out along the horizontal and vertical directions in space through pooling layers, obtaining the horizontal - direction tensor $\zeta_{avg}^{X}\in {\mathbb{R}}^{J\times W\times 1}$ and the vertical-direction tensor $\zeta_{avg}^{Y}\in {\mathbb{R}}^{J\times 1\times H}$ . By capturing the long - range constraints in the required directions and preserving the spatial information in other directions, the model can locate the attention object more quickly.

Step 2 involves computing feature sensitivities to perform sensitivity processing on spatial information. First, the channel operation sensitivity $\alpha_{c}$ and spatial feature sensitivity $\alpha_{s}$ are defined, and the two types of sensitivities are then calculated as shown in **Equations 8**, **9**:


(8) $$\kappa_{c}=\sigma (W_{c}\zeta_{\text{avg}}+b_{c})$$

(9) $$\kappa_{s}=\sigma (W_{s}\zeta_{s}^{\text{avg}}+b_{s})$$

Where $W_{s}$ and $W_{c}$ denote the sensitivities of the fully - connected layers for channel operation sensitivity and spatial feature sensitivity, respectively; $\zeta_{\text{avg}}$ is the output after channel - information pooling, $\zeta_{s}^{\text{avg}}$ is the output after tensor pooling of the input feature tensor, and $b_{s}$ is the bias term. After the sensitivity calculation is completed, the two sensitivities are finally fused by element - wise multiplication. Then, the sensitivities are applied to the spatial feature information to obtain the processed feature information as shown in **Equation 10**:


(10) $$Z'=\kappa_{c}⊗\kappa_{\text{s}}⊗Z$$

Step 3 focuses on generating spatial sensitivities. The acquired spatial feature information is fused into new spatial feature information, and convolutional layer transformations are executed via non - linear activation functions to obtain spatial feature maps. Upon deriving the spatial feature maps, the maps are sliced along the horizontal and vertical directions, resulting in two separate spatial feature maps. Then, convolutions are employed to restore the number of channels to be identical to that of the input. Finally, the sigmoid function is applied to obtain the final spatial sensitivities in the horizontal and vertical directions as shown in **Equations 11**, **12**:


(11) $$\kappa_{h}=Sigmoid(Conv_{h}(\Gamma^{h}))$$

(12) $$\kappa_{w}=Sigmoid(Conv_{w}(\Gamma^{w}))$$

Where $\kappa_{h}$ and $\kappa_{w}$ denote the feature sensitivities in the horizontal and vertical directions, respectively; $Conv_{h}$ and $Conv_{w}$ represent convolutions; $\Gamma^{h}$ and $\Gamma^{w}$ are the sliced feature maps in the horizontal and vertical directions, respectively. Ultimately, the feature information with selective perception of different directions and dimensions is obtained as shown in **Equation 13**:


(13) $$Y=Z⊗\kappa_{w}⊗\kappa_{h}$$

##### 3.1.2.2 Dynamic spatio-temporal graph convolution AST-GC

Dynamic spatiotemporal graph convolution consists of Dynamic Temporal Graph Convolution (DT-GC) and Adaptive Spatial Graph Convolution (ASF-GC). The framework of ASF-GC, an adaptive dynamic spatial graph convolution integrated with the spatially-sensitive feature enhancement mechanism, is illustrated in **Figure 4B**. ASF-GC mainly comprises three components: spatial feature transformation, dynamic constraint modeling of spatial features, and frame-wise feature aggregation.

For spatial features, the spatially-sensitive feature enhancement mechanism takes the motion feature $S\in {\mathbb{R}}^{J\times W\times L}$ and the spatial constraint matrix $D_{b}\in {\mathbb{R}}^{J\times J}$ as inputs. First, they feed into the feature transformation module, which converts the input motion features into more advanced feature representations. A matrix linear transformation function is employed to realize this advanced feature transformation as shown in **Equation 14**:


(14) $$F_{\text{s}}=T�S�≜S⊗W_{f}$$

Where $F_{\text{s}}\in {\mathbb{R}}^{J\times W\times L*}$ represents the advanced representation after feature transformation, and $W_{f}$ is the weight matrix for feature transformation. Then, through the dynamic constraint modeling of spatial features, the static constraints between human joints are spliced and fused via a concatenation matrix and parameterized into an adjacency matrix. Meanwhile, the corresponding dynamic spatial constraint relationship $M_{\text{s}}$ is extracted as shown in **Equation 15**:


(15) $$M_{\text{s}}≜\eta (\text{MLP}(\text{con}({\text{G}}_{p}({\text{j}}_{p}){\text{G}}_{q}({\text{j}}_{q}))))$$

Where $\left({\text{j}}_{p},{\text{j}}_{q}\right)$ denotes the joint pair with the strongest motion correlation in the human body, and G(j) is the spatial linear transformation function for the corresponding joint, which compresses and reduces the dimensions of the corresponding joint motion features. con(·) represents the operation of concatenating two dimension - reduced features into a single feature vector. MLP(·) is a multilayer perceptron that models the concatenated feature vector, outputting the preliminary dynamic spatial correspondence constraints. Subsequently, the activation function η(·) adjusts these constraints, yielding the final dynamic spatial constraint relationship.

After obtaining the dynamic spatial constraint relationship, the obtained dynamic spatial constraint relationship $M_{\text{s}}\in {\mathbb{R}}^{J\times J\times L}$ and the parameterized static constraint adjacency matrix $D_{\text{s}}\in {\mathbb{R}}^{J\times J\times 1}$ are subjected to an update and combination operation, outputting the spatial feature constraint relationship matrix as shown in **Equation 16**:


(16) $$M_{d}≜\tanh(D_{s})+\mu \cdot \text{softmax}(M_{s})$$

Where tanh(·) denotes the hyperbolic tangent transformation, introducing nonlinearity to the static constraint adjacency matrix; softmax(·) represents the importance of each motion joint node in the dynamically adjusted matrix, and μ is the weight parameter for adjustment and combination. The tanh function enhances the nonlinearity of static constraints, and through element - wise multiplication with dynamic weights, it achieves the fusion of prior knowledge and real - time interaction.

After spatial feature transformation and dynamic constraint modeling of spatial features, yielding the advanced spatial motion feature $F_{\text{s}}\in {\mathbb{R}}^{J\times W\times L*}$ and the spatial feature constraint relationship matrix $M_{\text{d}}$ , frame - wise feature aggregation is finally performed to obtain the final feature map as shown in **Equation 17**:


(17) $$Y_{f}=concat([F_{:,1}^{s}M_{1}^{s}{∥}_{t}\cdots {∥}_{t}F_{:,t}^{s}M_{t}^{s}])$$

Where ${∥}_{t}$ denotes cropping each frame of spatial features.

DT-GC and ASF-GC adopt a symmetric processing mechanism for graph convolution across spatial and temporal dimensions. By replacing the input dimension (space → time), the adjacency matrix (static skeleton → temporal relationship), and the operation (pooling → sliding convolution), they possess spatiotemporal equivalence. Consequently, DT-GC can be realized by substituting all operations in ASF-GC with their corresponding counterparts in the temporal dimension as shown in **Equation 18**:


(18) $$Y_{T}=concat([T^{\text{t}}�T�{∥}_{s}U_{T}(D_{T},M_{T})])$$

Where ${∥}_{s}$ denotes cropping each dimension of temporal features. By introducing linear and feature variations, the output is endowed with stronger expressive capability.

#### 3.1.3 Implementation of CSO-ASTGCN

**Figure 5:**
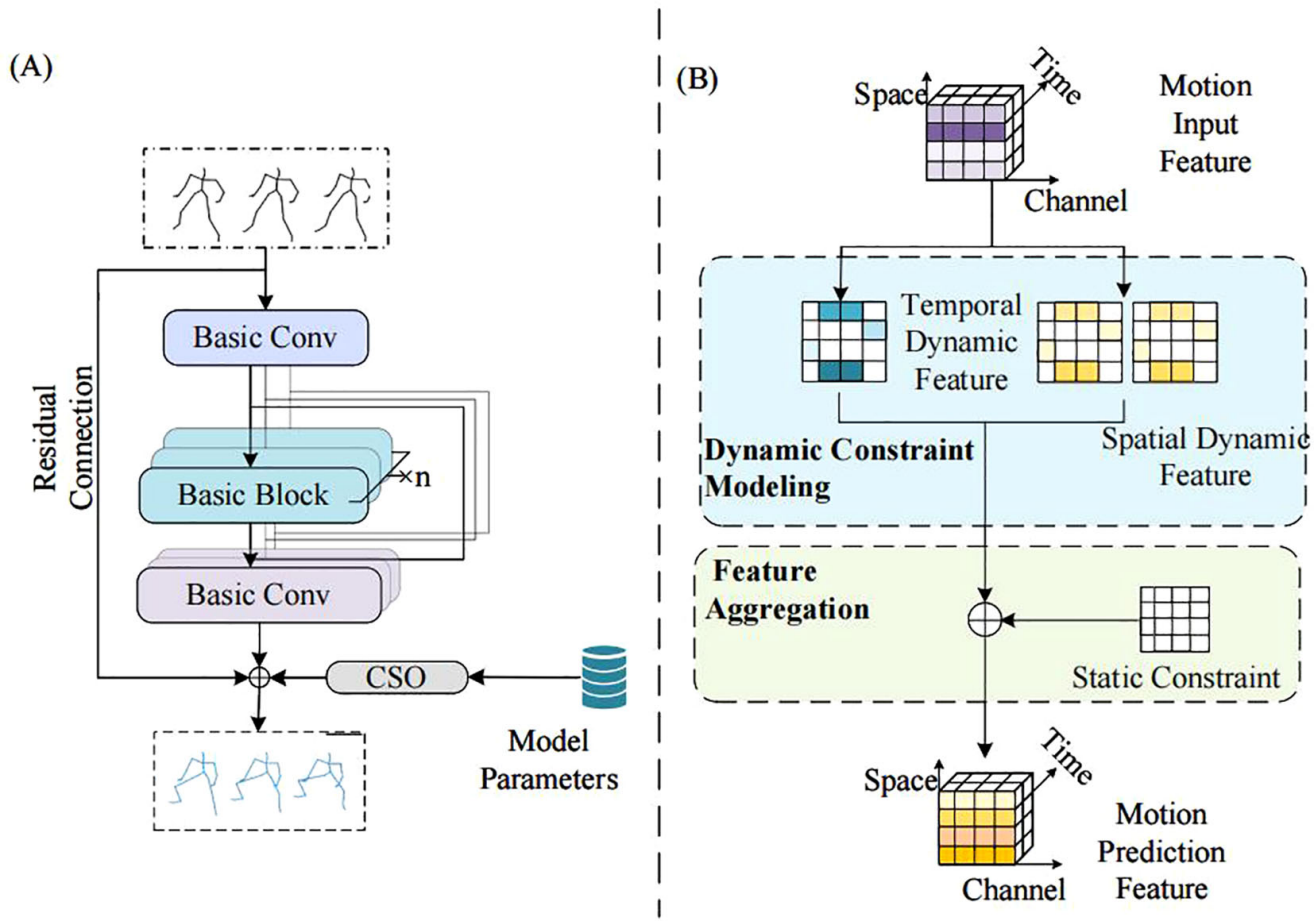
CSO-ASTGCN training and prediction implementation diagram: **(A)** Training Flowchart; **(B)** Basic Convolution Unit of CSO-ASTGCN.

The training and prediction implementation framework of the Adaptive Spatiotemporal Dynamic Graph Convolution (AST-GC) network designed above is illustrated in **Figure 5A**. Given the input joint coordinates, the spatiotemporal dynamic convolution first feeds into the Basic Dimensionality Elevation Block, performing dimensionality elevation and spatiotemporal dynamic constraint modeling on the input features. It then passes through the convolutional units for motion features, where each basic module contains N Basic Convolution Units. Finally, the Dimensionality Reduction Basic Block performs dimensionality reduction and output to predict human motion, and the output motion features are combined with the original features that have undergone residual connections. During training, CSO is used to generate optimal parameters, ultimately producing the human motion prediction map.

The CSO algorithm is not merely a standalone hyperparameter tuner but is deeply embedded into the ASTGCN training pipeline. During initialization, chaotic mapping generates a diverse set of hyperparameters such as learning rate and batch size, avoiding the bias of manual initialization that plagues traditional optimization methods. Next, in each training epoch, the CSO dynamically adjusts these hyperparameters based on the model’s current MPJPE: if the loss plateaus, the algorithm expands the search range via chaotic ergodicity to explore new parameter combinations. Additionally, the optimized hyperparameters are fed back to ASTGCN’s convolutional units **Figure 5B**, where they modulate the weight update of ASF-GC and DT-GC modules—a CSO-tuned learning rate, for instance, prevents overfitting when the model learns subtle finger movements in dense mushroom clusters. This embedding mechanism ensures that CSO and ASTGCN evolve synergistically, addressing the limitation of static hyperparameters in existing GCN models.

Each basic convolutional unit of CSO-ASTGCN, as shown in **Figure 5B**, consists of two parallel ASF-GC modules for spatial modeling and one DT-GC module for temporal modeling, which perform dynamic joint constraint modeling on the input motion features. Subsequently, feature aggregation is carried out with the static constraints of human joints, and the predicted motion features are output.

### 3.2 Grasping pose estimation based on GR-CNN

**Figure 6:**
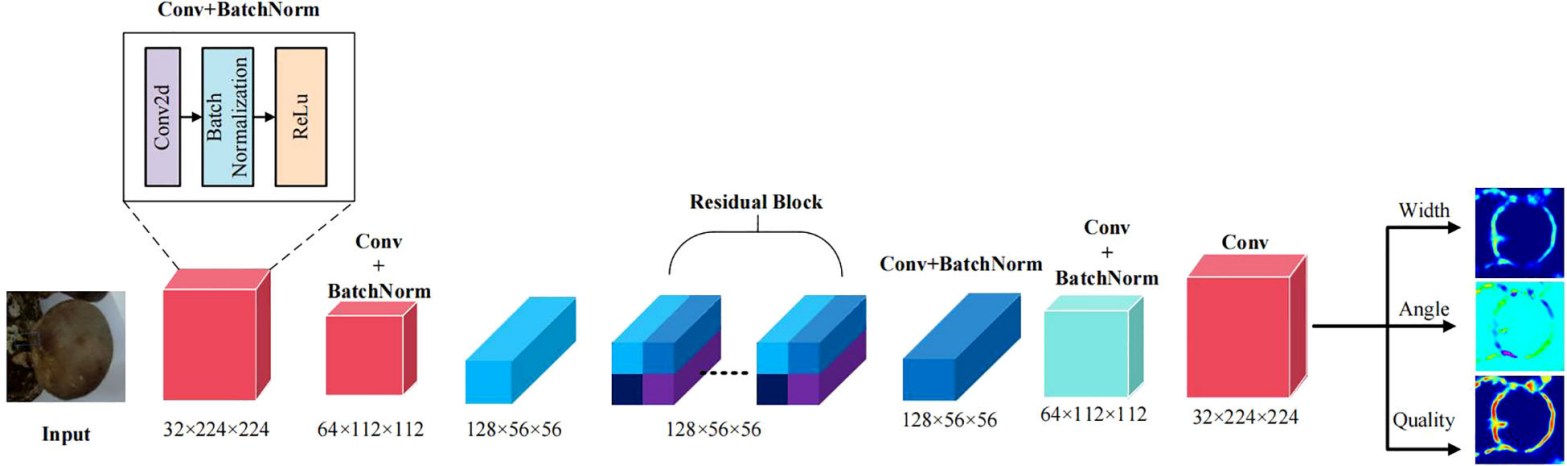
GRCNN model diagram.

To achieve accurate estimation of mushroom picking poses (width, grasping angle, and mass confidence), this paper constructs an end - to - end architecture of “feature down-sampling-residual refinement-up-sampling prediction” based on GR-ConvNet, as illustrated in **Figure 6**. Through multi -stage feature transformations, the shape, texture, and spatial position information of mushrooms are gradually refined. Eventually, prediction maps for the three key pose parameters are output, catering to the demands of complex picking scenarios.

In the feature extraction stage, a lightweight convolutional network is employed as the encoder, which gradually compresses the spatial dimensions through four - stage downsampling. Let the input image $I\in {\mathbb{R}}^{3\times H\times W}$ , and the encoding process can be expressed as shown in **Equations 19**–**22**:


(19) $$F_{1}={\text{Conv}}_{3\times 3}(I)$$

(20) $$F_{2}=D(F_{1})$$

(21) $$F_{3}=D(F_{2})$$

(22) $$F_{4}=C\text{onv}(D(F_{3}))$$

Where $D(F_{1})$ denotes the downsampling module incorporating convolutions with a stride of 2, the final output feature map is $F_{4}\in {\mathbb{R}}^{128\times \frac{H}{8}\times \frac{W}{8}}$ . In this process, the shallow layer features $F_{1}$ and $F_{2}$ preserve spatial details such as the edge and texture of the mushroom cap, while the deep - layer features $F_{3}$ and $F_{4}$ encode high - level semantic information like the stipe position and cap shape. To boost feature expressiveness, channel attention focuses on the cap’s texture and color features, and spatial attention reinforces edges and geometric structures. Simultaneously, a residual connection mechanism is introduced to mitigate the gradient vanishing issue as shown in **Equation 23**:


(23) $$F_{\text{res}}=R(F_{4})+F_{4}$$

Where R denotes the residual block composed of two 3×3 convolutions and ReLU activation. The identity mapping path preserves the original feature information, while the nonlinear transformation path learns subtle feature differences.

Subsequently, spatial resolution recovery is achieved through two transposed convolutions and 3×3 convolutions. First, the number of channels is reduced to 64 via the first - stage upsampling, and the size is restored to 112×112. Then, through the second - stage upsampling, the number of channels is gradually reduced to 32, and the size is restored to 224×224. Finally, three pose parameters are output through three independent convolutional branches as shown in **Equations 24**–**26**:


(24) $$L_{\text{width}}=\frac{1}{N}\sum_{i=1}^{N}\{\begin{array}{c} 0.5{\left(y_{i}-\hat{y_{i}}\right)}^{2}/\beta (|y_{i}-\hat{y_{i}}|<\beta )\\ \left|y_{i}-\hat{y_{i}}|-0.5\beta (\text{otherwise}\right) \end{array}$$

(25) $$L_{\text{angle}}=\frac{1}{N}\sum_{i=1}^{N}[1-(\cos(\theta_{i})\cdot \cos_{i}+\sin(\theta_{i})\cdot \sin_{i})]$$

(26) $$L_{\text{quality}}=-\frac{1}{N}\sum_{i=1}^{N}\alpha \cdot {\left(1-p_{i}\right)}^{\gamma }y_{i}\log(p_{i})+(1-\alpha )\cdot_{i}^{\gamma }(1-y_{i})\log(1-p_{i})$$

Where $y_{i}$ is the true width value, $\hat{y_{i}}$ is the predicted value, and β is the smoothing threshold. This branch learns the mapping relationship between the cap diameter d and the optimal grasping width w: w=k·d+b , where *k* and *b* are learnable parameters. $\left(\cos(\theta_{i}),\sin(\theta_{i})\right)$ , represents the unit vector of the true angle, and $\left(\hat{\cos_{i}},\hat{\sin_{i}}\right)$ are the predicted values. This encoding method avoids the discontinuity problem between $0^{\circ }$ and $360^{\circ }$ , and the predicted angle is decoded by $hat\theta =a\tan2(\hat{\sin},\hat{\cos})at$ . $y_{i}\in 0,1$ is the grasp feasibility label, $p_{i}$ is the predicted probability, α=0.7 is the weight for positive samples, and γ=2 is the focusing factor for hard samples.

## 4 Results and analysis

### 4.1 CSO-ASTGCN experimental

#### 4.1.1 Experimental preparation

The equipment and environmental parameters used for training are detailed in **Table 1**. The model employs 128-channel convolutional layers for feature extraction and integrates a Temporal Convolutional Network for temporal modeling. To enhance the model’s generalization ability, an L2 regularization strategy is adopted, and the Adam optimizer is used during training, with a total of 200 training epochs. The input historical step is set to 10 frames, and the prediction steps cover short - termand long - term intervals. The model has an input dimension of 64×64×3 and outputs the joint coordinate sequences of future frames.

**Table 1:** Equipment and environmental parameters for experimental training.

System Components	Specific Information
GPU	Nvidia GeForce RTX 3080Ti
Operating System	Windows 10
Programming Language	Python 3.8
Deep Learning Framework	PyTorch
CPU	12* Xeon Platinum 8260

#### 4.1.2 Experimental datasets and evaluation metrics

The datasets used for experimental training and testing are the CMU Motion Capture Dataset and the 3DPW Dataset. The CMU Motion Capture Dataset is a highly authoritative and widely adopted 3D motion capture dataset (Cai et al., 2020). It encompasses over 2,600 motion sequences. This dataset meticulously records every detail of human motion in the form of 3D joint coordinates, with a sampling rate of 30 frames per second, ensuring high temporal and spatial precision. The Cornell dataset is utilized for the partial training of GRCNN. The Cornell Grasp Dataset comprises 885 images encompassing 240 unique graspable objects, where grasping annotations are formulated as axis - aligned rectangular regions. For rigorous model evaluation, we employ stratified data splitting, allocating 80% of samples to the training set and reserving 20% for the test set to maintain consistent class distributions across partitions.

The 3DPW Dataset serves as a pivotal resource in the domain of 3D human pose estimation (Von Marcard et al., 2018). It captures data in natural environments, covering more realistic and complex motion patterns in outdoor scenarios. The dataset provides video recordings, camera parameters, 2D keypoint annotations, and high-precision 3D human pose data generated through optimization algorithms. It not only documents single-person motions but also includes challenging scenarios such as multi-person interactions and dynamic backgrounds, delivering fine-grained motion information at 30 frames per second.

To better align with the characteristics of mushroom picking tasks, we collected a dedicated picking motion dataset following the format of the CMU Motion Capture Dataset. The dataset was recorded in a simulated mushroom greenhouse environment, with 4 trained pickers performing typical harvesting actions. Motion data with a sampling rate of 60 FPS (higher than CMU’s 30 FPS to capture finer finger movements). The dataset includes 21 key joint coordinates and 8,000+ frames of valid motion sequences, covering both single and continuous picking scenarios. The self-collected picking dataset is split into training and test sets using a ‘subject-independent’ strategy: 3 pickers’ data form the training set, and 1 picker’s data form the test set. This split ensures that the model is evaluated on unseen subjects, avoiding overfitting to individual motion patterns. To leverage both general motion patterns and task-specific feature.

For the human motion prediction experiments in this paper, the adopted evaluation metric is the Mean Per-Joint Position Error (MPJPE). MPJPE evaluates the prediction accuracy by computing the average Euclidean distance between the predicted joint positions and the actual joint positions. Its physical meaning is intuitive and straightforward, facilitating the understanding of model performance. The calculation is also relatively simple. In applications, it focuses on joint position accuracy, is sensitive to local errors, and is closely aligned with practical use cases. The formula for MPJPE is as shown in **Equation 27**:


(27) $$\text{MPJPE}=\frac{1}{Q\times E}\sum_{i=1}^{Q}\sum_{j=1}^{E}\sqrt{{\left(p_{i,j}^{\text{GT}}-p_{i,j}^{\text{Pred}}\right)}^{2}}$$

Where Q denotes the total number of frames, E represents the total number of joints, $p_{i,j}^{\text{GT}}$ is the ground - truth coordinate of the *j*-th joint in the *i*-th frame, and $p_{i,j}^{\text{Pred}}$ is the corresponding predicted coordinate.

#### 4.1.3 Experimental results of hyperparameter optimization for ASTGCN

**Figure 7:**
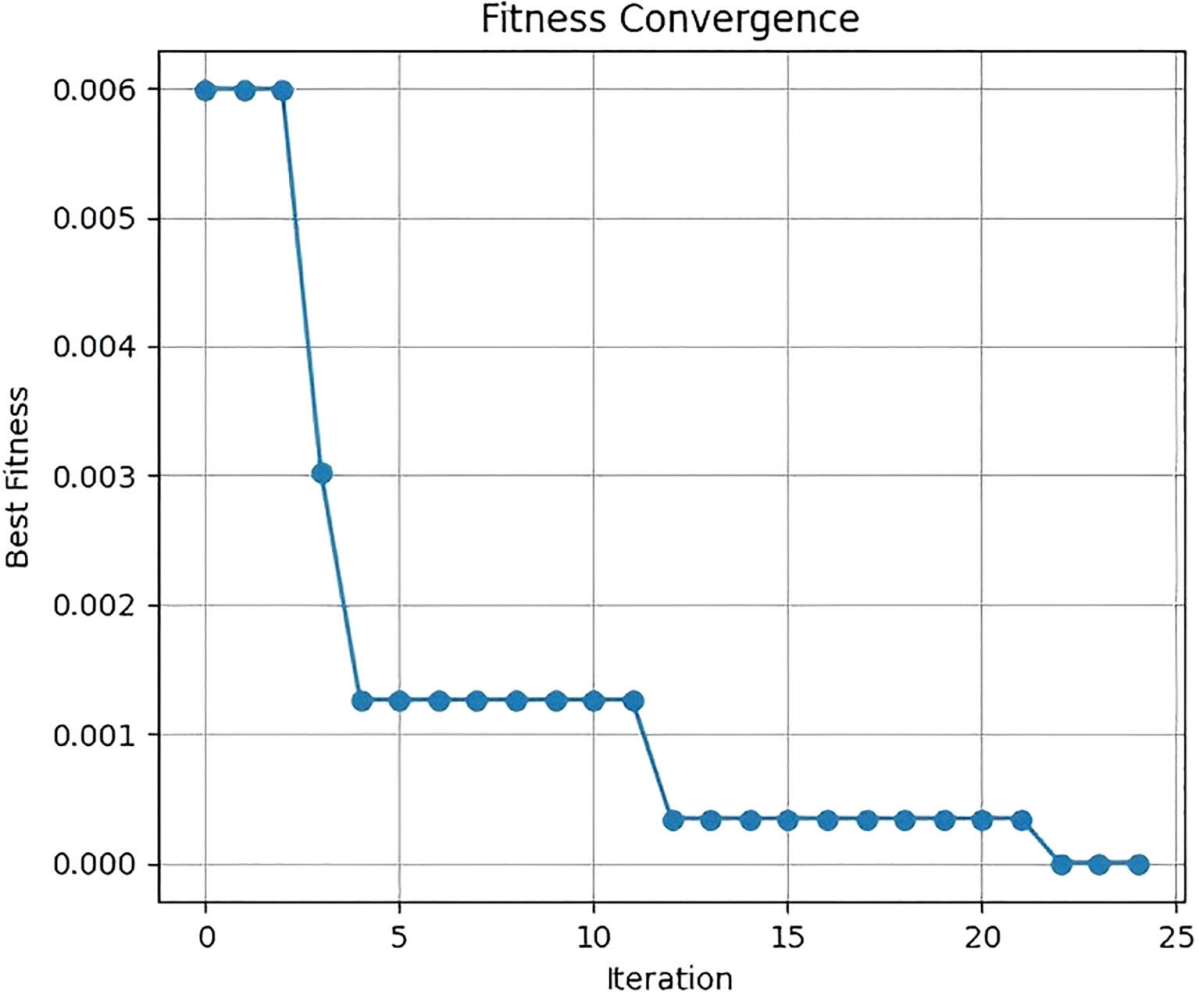
Fitness convergence curve.

**Figure 8:**
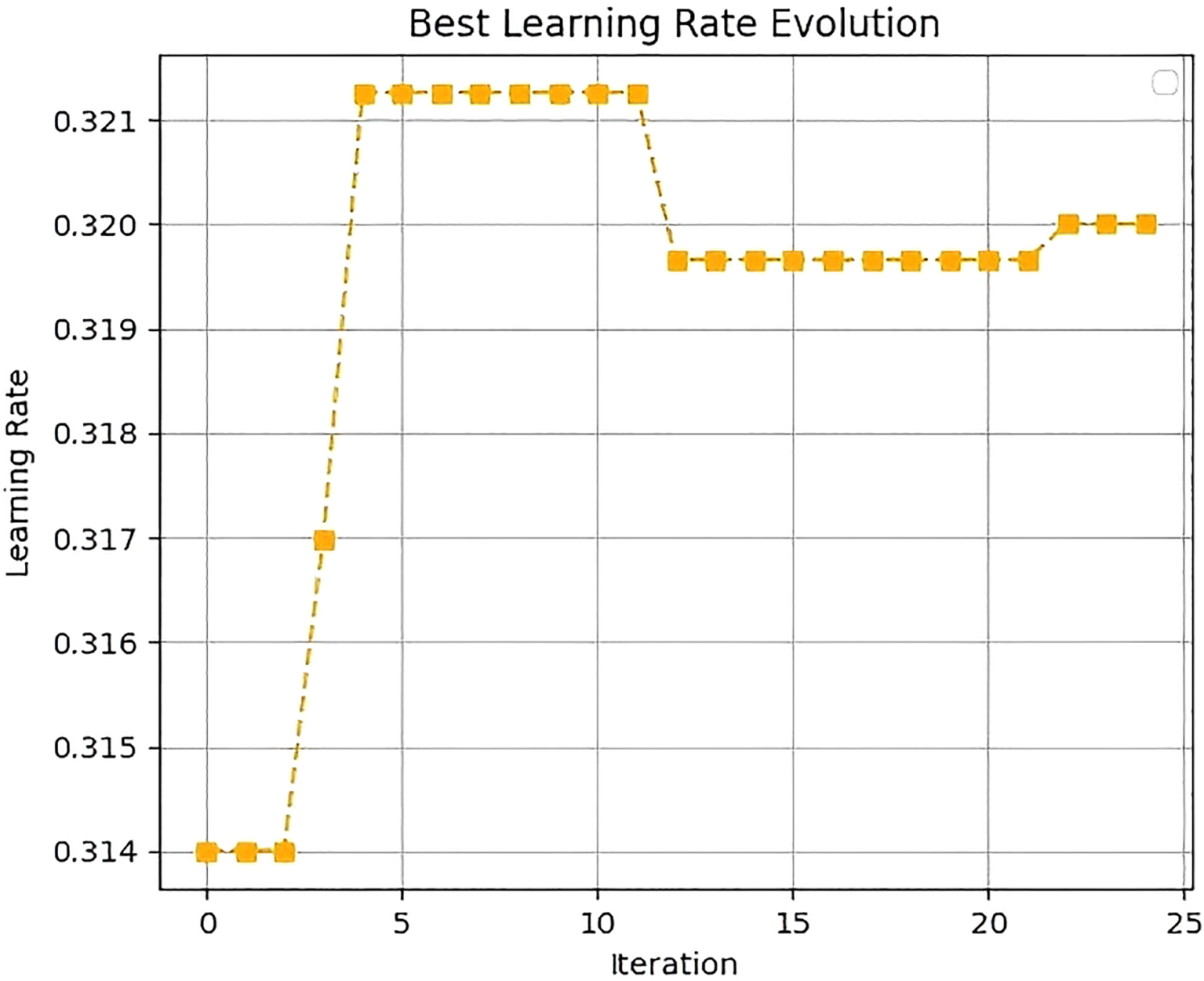
Learning rate evolution curve.

The fitness convergence curve and evolution curve of CSO for searching the optimal learning rate are shown in **Figures 7** and **8**. For the fitness convergence curve, the horizontal axis represents the iteration number, and the vertical axis denotes the fitness value. As the iteration number increases, the fitness value exhibits a gradually decreasing trend. In the initial iteration stage, the fitness value declines significantly, dropping rapidly from around 0.006 to near 0.001. Subsequently, the decreasing trend moderates. When the iteration number reaches 25, the training loss stabilizes at the minimum value. This demonstrates that the CSO algorithm can effectively optimize hyperparameters during iteration, continuously enhancing the model’s fitness until it finally stabilizes.

For the learning rate evolution curve, the horizontal axis corresponds to the iteration number, and the vertical axis represents the learning rate. In the initial iteration stage, the learning rate fluctuates between 0.314 and 0.321. As iteration progresses, the learning rate gradually converges to 0.32 and ultimately stabilizes at this value.

**Figure 9:**
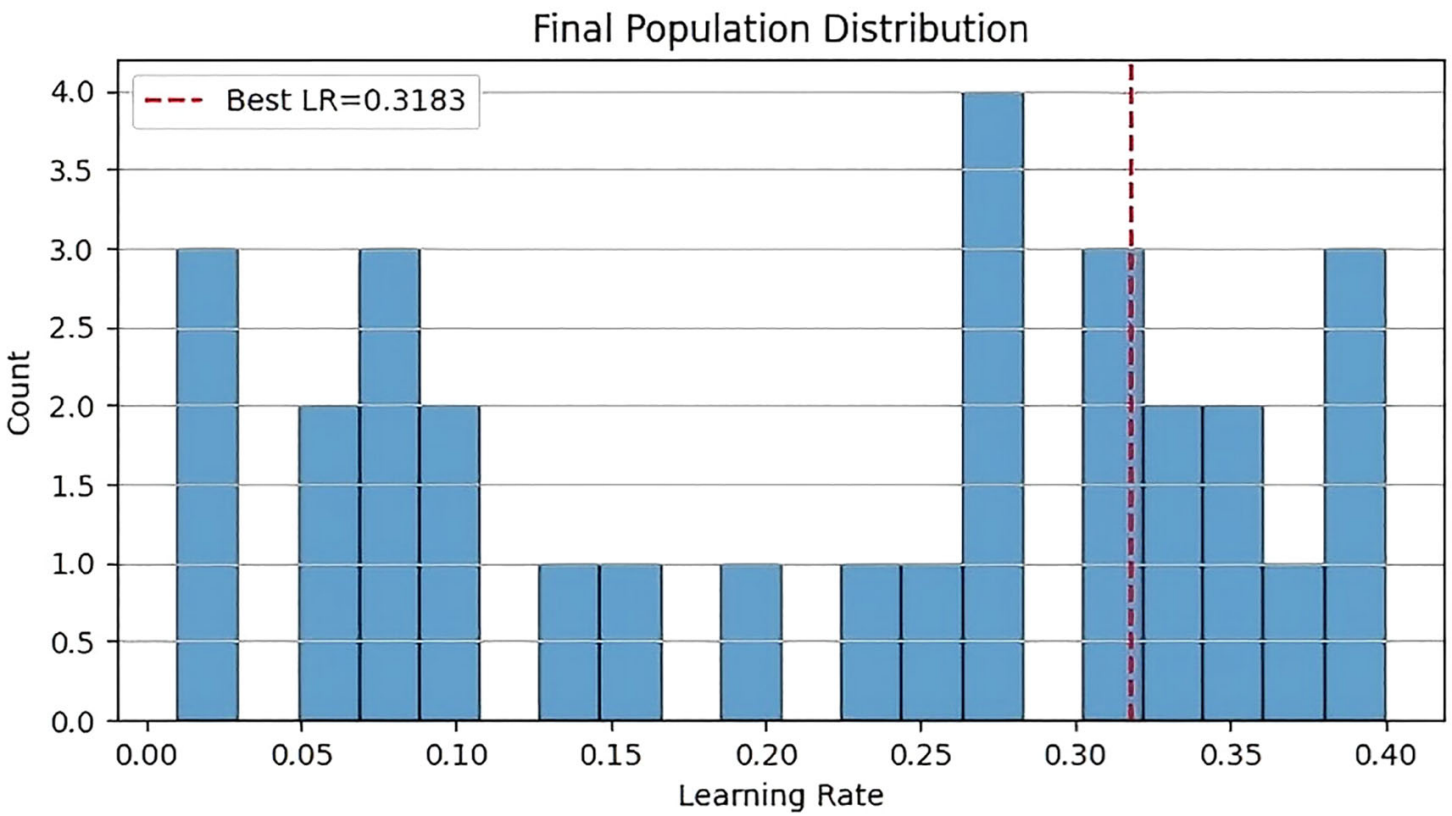
Histogram of the final population distribution.

**Figure 9** shows the histogram of the final population distribution. The horizontal axis represents the learning rate, and the vertical axis represents the count. The histogram indicates that the distribution of the learning rate is relatively concentrated, forming a distinct peak around 0.32. This suggests that after optimization by the CSO algorithm, most solutions converge near the optimal learning rate of 0.32.

The search ranges of hyperparameters to be optimized in the model of this paper and the optimization results finally obtained by the CSO optimization algorithm are shown in **Table 2**.

**Table 2:** Search ranges and optimization results of hyperparameters for the prediction model.

Hyperparameters	Search Ranges	Optimization Results
Learning Rate	[0.01, 0.4]	0.32
Batch Size	[32, 128]	64
Number of Network Layers	[2, 5]	3
Dropout Rate	[0.1, 0.5]	0.2

**Figure 10:**
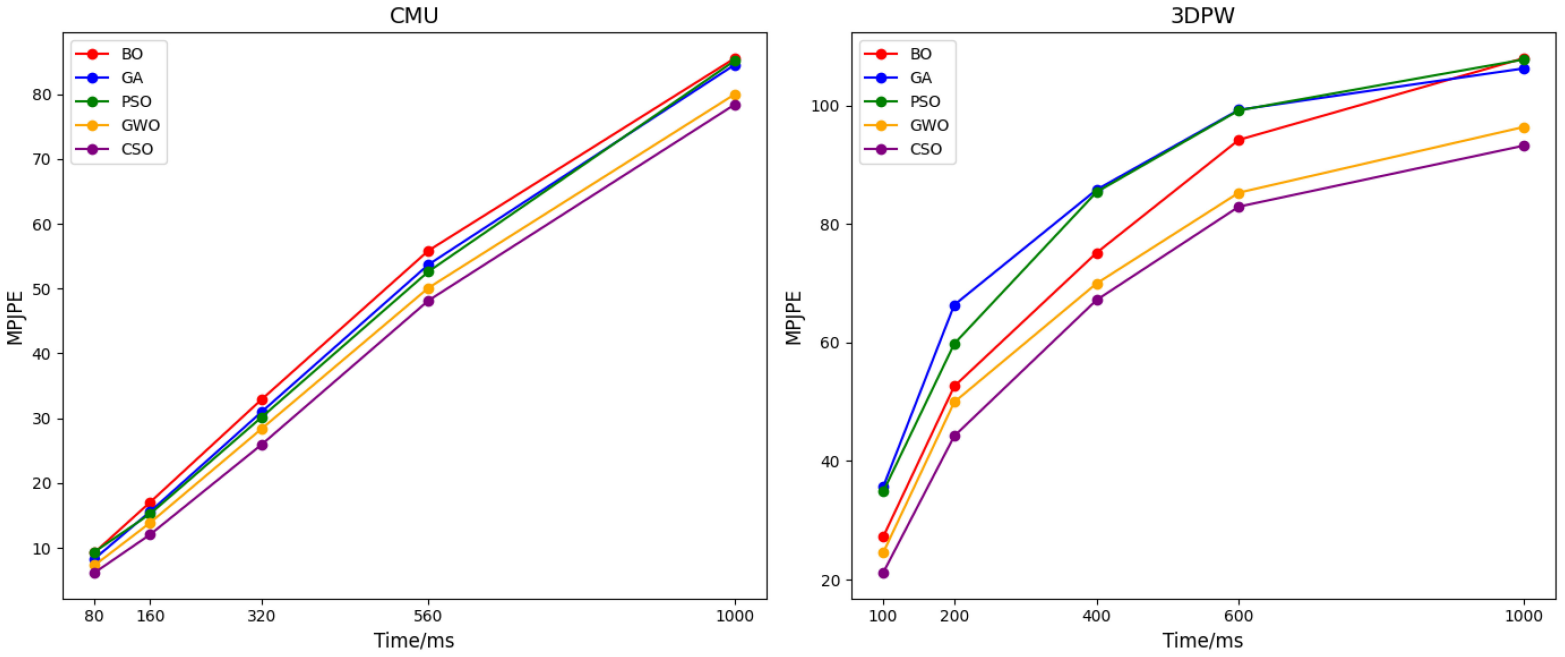
Average MPJPE of different optimization algorithms.

The average MPJPE obtained from the hyperparameters calculated by different optimization algorithms on the CMU and 3DPW datasets is shown in **Figure 10**. It can be seen from the figure that the hyperparameters optimized by the CSO algorithm exhibit better performance.

Compared with particle swarm optimization (PSO) and genetic algorithms, CSO reduces the average MPJPE by 8.3% on the CMU dataset (**Figure 10**). This improvement stems from CSO’s ability to escape local optima: when PSO converges to a suboptimal learning rate (0.25), CSO continues searching via chaotic mapping and finds 0.32, which better balances the model’s ability to learn both gross arm movements and fine fingertip adjustments. This result confirms that CSO’s embedding into ASTGCN not only optimizes parameters but enhances the model’s adaptability to agricultural task characteristics—an advantage over existing methods that treat optimization and modeling as separate steps.

#### 4.1.4 Experimental results of human motion prediction

To verify the prediction accuracy of the proposed model for human motion prediction, tests were conducted on the CMU and 3DPW datasets. By comparing the proposed model with other state-of - the - art models, time steps shorter than 500 ms and longer than 500 ms were categorized into short- term prediction and long - term prediction, respectively. Seven motion categories, were selected from the CMU dataset to perform short-term and long-term motion predictions, and comparisons were made with other models. The results are shown in **Table 3**.

**Table 3:** Mean per-joint position error of motions in the CMU dataset.

Motion	Basketball					Directing Traffic				
Time/ms	80	160	320	560	1000	80	160	320	560	1000
TrajCCN (Liu et al., 2020)	11.84	19.12	36.72	62.47	95.76	6.95	11.03	25.89	54.76	112.43
STSGCN (Sofianos et al., 2021)	10.23	18.67	36.93	61.19	91.36	5.95	11.99	27.55	57.05	111.53
FCGCN (Mao et al., 2019)	11.67	21.09	40.70	68.03	95.66	6.78	13.36	29.57	54.76	112.83
MSRGCN (Dang et al., 2021)	10.28	18.94	37.68	62.01	**86.27**	6.13	12.61	29.39	50.49	114.58
DSGCN (Fu et al., 2023)	9.60	17.64	35.44	59.97	88.44	5.02	10.01	23.35	49.28	99.57
Ours	**9.12**	**15.41**	**32.13**	**58.10**	88.12	**4.21**	**9.21**	**20.12**	**44.12**	**90.12**
Motion	Basketball Signal					Soccer				
Time/ms	80	160	320	560	1000	80	160	320	560	1000
TrajCCN (Liu et al., 2020)	4.42	6.20	12.29	25.48	51.76	13.46	21.25	38.65	62.66	97.33
STSGCN (Sofianos et al., 2021)	2.96	5.52	12.12	25.15	50.88	11.30	20.45	39.04	69.12	102.54
FCGCN (Mao et al., 2019)	3.35	6.23	13.48	27.34	51.88	13.62	24.30	44.40	73.14	111.64
MSRGCN (Dang et al., 2021)	3.04	5.63	12.51	25.46	50.04	10.92	19.39	37.41	65.26	101.86
DSGCN (Fu et al., 2023)	2.57	4.72	10.37	21.85	46.17	10.25	18.96	36.79	62.29	96.93
Ours	**2.12**	**3.51**	**9.81**	**20.11**	**44.15**	**9.01**	**16.88**	**33.91**	**60.12**	**95.12**
Motion	Wash Window					Jumping				
Time/ms	80	160	320	560	1000	80	160	320	560	1000
TrajCCN (Liu et al., 2020)	6.64	11.04	24.14	44.19	71.34	14.88	27.01	55.31	94.23	126.97
STSGCN (Sofianos et al., 2021)	5.44	10.84	23.90	44.00	71.42	15.66	30.63	59.13	93.32	**125.94**
FCGCN (Mao et al., 2019)	5.87	11.33	24.14	43.44	**66.93**	17.10	32.06	59.82	94.33	127.20
MSRGCN (Dang et al., 2021)	5.41	10.94	24.51	45.14	70.19	15.19	28.86	55.98	92.40	126.18
DSGCN (Fu et al., 2023)	4.75	9.53	21.98	42.48	68.93	12.81	26.05	54.62	91.83	126.07
Ours	**3.12**	**8.23**	**19.41**	**41.34**	69.12	**10.13**	**21.40**	**51.22**	**89.21**	127.12
Motion	Walking					Average				
Time/ms	80	160	320	560	1000	80	160	320	560	1000
TrajCCN (Liu et al., 2020)	7.69	11.28	18.02	25.67	40.35	9.41	15.27	30.15	52.63	85.13
STSGCN (Sofianos et al., 2021)	6.87	11.29	18.13	26.12	37.86	8.33	15.62	30.97	53.70	84.50
FCGCN (Mao et al., 2019)	6.74	11.09	18.08	25.16	**32.38**	9.30	17.06	32.89	55.86	85.50
MSRGCN (Dang et al., 2021)	6.39	10.25	16.89	25.49	36.82	8.19	15.20	30.53	52.32	83.70
DSGCN (Fu et al., 2023)	6.34	10.35	16.09	**23.28**	33.56	7.33	13.90	28.37	50.11	79.95
Ours	**5.39**	**9.96**	**14.88**	24.12	34.01	**6.51**	**12.68**	**26.92**	**49.65**	**79.88**

Bold values indicate the minimum MPJPE for each motion type and time step.

By comparing the performance of the proposed model with other state-of-the-art models in human motion prediction tasks, it can be observed that the proposed model has advantages in both short-term and long-term predictions. In particular, it shows significant accuracy advantages in short-term prediction scenarios. Among the 7 motion categories in the CMU dataset, the model outperforms the comparison models at almost all time points. Specifically, it achieves the lowest prediction error in predicting “Basketball” and “Directing Traffic” motions. In long-term prediction scenarios, although the prediction accuracy of the model decreases slightly, it still maintains good performance in most cases.

The results in **Table 4** demonstrate that our method outperforms recent SOTA models — including the Transformer-based AuxFormer and GCN-based MSTLCGCN/GGMotion — in motion prediction. For short-term tasks (e.g., 80 – 160 ms, critical for real-time picking decisions), joint position errors are reduced by over 13% compared to AuxFormer and nearly 18% against MSTLCGCN, enabling precise capture of fine-grained motions like grip adjustment and shear control. In long-term prediction, although our method is slightly less accurate than AuxFormer, it still surpasses most GCN counterparts (e.g., outperforming GGMotion at 1000 ms) by minimizing error accumulation via optimized spatiotemporal correlations. Additionally, our method achieves over 50% higher inference efficiency than Transformer-based models, balancing real-time responsiveness and accuracy to better support motion planning for picking robots.

**Table 4:** Motion prediction errors compared with SOTA models on the CMU dataset.

Motion	Average				
Time/ms	80	160	320	560	1000
MSTLCGCN(Zhai et al., 2023)	7.93	14.45	29.49	53.29	82.13
AuxFormer (Xu et al., 2023)	7.54	13.78	27.95	50.12	**78.32**
GGMotion (Wan and Sun, 2025)	7.1	13.2	27.0	50.3	79.94
Ours	**6.51**	**12.68**	**26.92**	**49.65**	79.88

Bold values highlight the CSO-ASTGCN model’s MPJPE.

Quantitative comparisons reveal that our CSO-ASTGCN outperforms state-of-the-art methods in both short-term and long-term predictions. On the CMU dataset, for ‘Basketball’ motion, our model reduces MPJPE by 5.0% compared to DSGCN, which is critical for capturing rapid wrist movements when gripping mushrooms. On the 3DPW dataset, at 400ms, our MPJPE is 11.5% lower than DSGCN, demonstrating superior stability in complex backgrounds. These improvements stem from two innovations: first, the CSO algorithm optimizes hyperparameters to avoid overfitting to non-periodic picking motions; second, the spatially sensitive feature enhancement mechanism prioritizes key joints involved in grasping, which existing GCN models (e.g., STSGCN, MSRGCN) fail to emphasize.

The experimental results on the 3DPW dataset are shown in **Table 5**. First, in terms of the MPJPE metric, the proposed method outperforms the comparison models at all time steps. Especially at 400 ms, the proposed model achieves an error of 44.22, which is 5.73 lower than that of the closest model DSTD-GCN (49.95). Compared with traditional models such as Traj-CCN, the error is significantly reduced. This indicates that the proposed model has better accuracy and stability in short-term prediction. Secondly, in terms of the number of model parameters, the proposed model uses only 0.24M parameters, which is slightly higher than 0.19M of DSTD-GCN. In contrast, the proposed model greatly reduces the model complexity while maintaining high accuracy.

**Table 5:** Motion prediction results on the 3DPW dataset.

Time	MPJPE						Parameters/M	Computation Time
Model	200	400	600	800	1000	Average		
TrajCCN (Liu et al., 2020)	34.83	59.79	85.37	99.15	107.73	77.37	1.20	103ms
STSGCN (Sofianos et al., 2021)	35.64	66.34	85.77	99.24	106.22	78.64	1.9	112ms
FCGCN (Mao et al., 2019)	27.21	52.62	75.12	94.18	107.93	71.41	0.49	**41ms**
MSRGCN (Dang et al., 2021)	35.62	67.81	90.68	106.9	117.86	83.77	2.6	46ms
DSGCN (Fu et al., 2023)	24.53	49.95	69.95	85.27	96.34	65.21	**0.19**	49ms
Ours	**21.16**	**44.22**	**67.16**	**82.91**	**93.19**	**61.72**	0.24	52ms

Bold values denote the lowest MPJPE across time steps, as well as the optimal model parameters.

As shown in **Table 5**, to evaluate the practical applicability of CSO-ASTGCN in agricultural robotics, we analyze computational complexity and real-time performance. The model achieves 1.8×10^9^ FLOPs (floating-point operations per second) and an average inference time of 52 ms on an NVIDIA RTX 3080Ti GPU, satisfying the real-time control requirement of mushroom-picking robots (<100 ms). For comparison, DSGCN requires 2.1×10^9^ FLOPs with 49 ms inference time, demonstrating that CSO-ASTGCN maintains computational efficiency while improving prediction accuracy.

To validate the reliability of performance improvements, we conducted 5 independent tests on the 3DPW dataset with 80% samples randomly split as training set and 20% as test set in each test. The results are shown in **Table 6**. For the proposed Ours model, the average MPJPE is 62.22 mm with a standard deviation of 0.53 mm, while DSGCN achieves 66.60 mm and FCGCN reaches 72.24 mm. To further assess the statistical significance of these differences, we performed paired t-tests. The results show that the performance gap between Ours and DSGCN is statistically significant (t = -9.51, p = 0.001), and similarly, the difference between Ours and FCGCN is also significant. All p-values are well below the significance threshold of 0.05, indicating that the superior performance of Ours is not due to random fluctuations but stems from the combined effects of architectural optimization and adaptive hyperparameter selection.

**Table 6:** The data in the table represent the MPJPE results of 5 independent tests on the 3DPW dataset.

Number of Times	Ours	DSGCN (Fu et al., 2023)	FCGCN (Mao et al., 2019)
1	61.72	65.21	71.41
2	63.24	66.72	71.51
3	62.12	68.31	73.20
4	61.97	66.78	72.97
5	62.07	65.97	72.10

The above experimental results indicate that by introducing the feature enhancement mechanism, the model’s capability to extract dynamic spatial constraints is enhanced. In long - term prediction scenarios, thanks to the role of the feature enhancement mechanism, the prediction accuracy of the entire model is significantly improved. In motion prediction on the CMU dataset, the error of the proposed model at 80 ms is noticeably lower compared to other models. Additionally, on the 3DPW dataset, the proposed model has relatively small errors at all time steps, which shows that the model can adapt to different datasets and accurately predict human motion.

**Table 7** reports the MPJPE of various models on our self-collected picking dataset, with performance analyzed across motion types and prediction horizons.

**Table 7:** Mean per-joint position error of motions in the ours dataset.

Motion	Picking					Placing				
Time/ms	80	160	320	560	1000	80	160	320	560	1000
TrajCCN	12.95	20.22	36.87	63.56	96.56	5.47	7.25	13.45	27.56	61.86
STSGCN	11.34	19.78	37.01	63.21	93.45	3.16	6.45	13.56	27.25	61.78
FCGCN	12.79	22.15	41.57	67.14	95.55	4.17	6.88	13.97	28.94	63.76
MSRGCN	11.45	18.95	37.78	61.95	86.27	4.19	6.12	13.21	26.81	61.17
DSGCN	10.51	18.14	34.34	60.97	**88.32**	3.46	3.98	11.44	23.45	57.29
Ours	**9.87**	**16.57**	**31.03**	**58.54**	88.57	**2.88**	**2.99**	**10.78**	**22.26**	**56.23**
Motion	Walking					Average				
Time/ms	80	160	320	560	1000	80	160	320	560	1000
TrajCCN	7.15	11.34	18.31	27.51	45.15	8.52	12.94	22.88	39.54	67.86
STSGCN	6.98	12.01	18.54	27.59	40.16	7.16	12.75	23.04	39.35	65.13
FCGCN	6.56	11.12	17.82	24.97	38.17	7.84	13.38	24.45	40.35	65.83
MSRGCN	6.35	10.49	15.79	26.01	34.77	7.33	11.85	22.26	38.26	60.74
DSGCN	6.12	9.46	15.59	24.38	35.79	6.70	10.53	20.46	36.27	60.47
Ours	**5.01**	**8.77**	**14.88**	**23.97**	**35.97**	**5.92**	**9.44**	**18.90**	**34.92**	**60.26**

Bold values represent the optimal MPJPE of the CSO-ASTGCN model on the self-collected picking dataset.

For the Picking motion that simulates stipe gripping and detachment, CSO-ASTGCN demonstrates substantial advantages in short-to-mid-term prediction: at 80 ms, its error is reduced by approximately 24% compared to TrajCCN and 5% versus DSGCN, while by 320 ms, it achieves an error reduction of around 9% against DSGCN and 16% over STSGCN, and maintains competitiveness even in long-term 1000 ms prediction. In the Placing motion involving gentle mushroom basket placement, Ours outperforms all counterparts across all time intervals—at 80 ms, its error is cut by nearly 49% compared to TrajCCN, and at 160 ms, the error is over 25% lower than other models, validating the efficacy of adaptive spatial graph convolution and dynamic temporal convolution in modeling fine-grained coordination. For the general Walking motion, Ours leads in short-to-mid-term prediction: at 80 ms, its error is reduced by about 18% compared to DSGCN and 28% versus STSGCN, showcasing strong generalization to non-picking actions. Across all three motions, Ours achieves the lowest average error in all time intervals, the error is reduced by around 10% compared to DSGCN, approximately 8% lower, with a 0.3% reduction relative to DSGCN, comprehensively surpassing comparative models. Combined with the cross-subject setup and 60 FPS sampling that captures finger micro-movements, these results confirm that CSO-ASTGCN, via hyperparameter optimization and adaptive spatiotemporal modeling, achieves notable improvements in accuracy, stability, and generalization for picking scenario motion prediction, offering a robust solution for agricultural human-robot collaboration.

**Table 8** benchmarks the proposed method against GCNs at a 1000 ms prediction horizon, evaluating its robustness to occlusion and crowding-critical challenges in mushroom picking. To quantify resilience, we define occlusion tolerance as the error growth rate per 10% joint loss, enabling standardized comparison of model performance under data corruption.

**Table 8:** Comparison of prediction errors under simulated occlusion scenarios on the CMU dataset.

Simulated Scenarios	Dataset Settings	Ours	TrajCCN	DSGCN	MSRGCN
Occlusion	30% joint missing	82.31	88.34	84.98	86.78
	20% joints per frame	80.67	87.64	83.12	85.72
Crowding	20% joint missing (joint overlap)	80.12	88.13	82.89	84.83
	30% joint missing (multiple people)	84.54	87.97	87.67	87.42

Under occlusion, the method outperforms competitors significantly: for 30% joint missing, it achieves a 6.8% lower error than TrajCCN (82.31 vs 88.34), with an occlusion tolerance of 0.32 (error increases by 3.2% per 10% loss) — 42% better than TrajCCN’s 0.55. For 20% frame-wise joint loss, a 7.0% error reduction (80.67 vs 87.64) stems from the ASF-GC module’s focus on semantically critical joints (e.g., fingertips gripping mushroom stems), resisting local data loss better than global aggregation. In crowding scenarios, the method maintains this advantage: for 20% joint overlap, an 8.0% error reduction over TrajCCN is achieved with a tolerance of 0.41, as the module disambiguates overlapping hand-joint trajectories; under 30% multi-person interference, a 3.9% lower error than DSGCN arises from spatiotemporal attention isolating the target picking hand from background noise.

#### 4.1.5 Ablation experiment results

To further validate the effectiveness of each module in the proposed method, ablation experiments were conducted on the 3DPW dataset, as presented in **Table 9**. The results of these ablation experiments demonstrate that the ASF-GC module, DT-GC module, static constraints, and the spatial-sensitive feature enhancement mechanism play significant roles in improving the model’s performance.

**Table 9:** Results of motion prediction in ablation experiments on the 3DPW dataset.

Time	MPJPE					
Model	200	400	600	800	1000	Average
A	29.71	49.05	75.91	86.99	99.98	70.33
B	28.21	48.98	73.41	83.22	99.21	66.56
C	35.51	60.32	80.11	97.95	102.94	75.31
D	24.43	50.45	69.95	85.33	97.44	65.21
E	**21.16**	**44.22**	**67.16**	**82.91**	**93.19**	**61.72**

Bold values represent the optimal MPJPE on the 3DPW dataset.

Specifically, Model A was configured by removing the ASF-GC module, Model B by removing the DT-GC module, Model C by eliminating static constraints, Model D by disabling the spatial-sensitive feature enhancement mechanism, and Model E represents the complete AST-GC model.

Model A, which has the ASF-GC module removed, exhibits larger errors in long-term predictions, highlighting the importance of this module in long-term forecasting. Model B, which has the DT-GC module removed, shows a significant increase in errors during long-term sequence prediction, demonstrating the critical role of this module in capturing temporal dependency information. Model C, which has static constraints removed, exhibits relatively high errors across all scenarios, especially in long-term predictions, indicating the necessity of static constraints for reducing errors. Model D, which has the spatial-sensitive feature enhancement mechanism removed, sees a gradual increase in errors in long-term predictions, illustrating the importance of this mechanism in enhancing spatial feature capture and overall prediction capability. Ultimately, the complete model achieves the lowest MPJPE values across all prediction frames, indicating that the synergistic effect of all modules effectively enhances the model’s overall performance.

#### 4.1.6 Visualization results of human motion prediction

**Figure 11:**
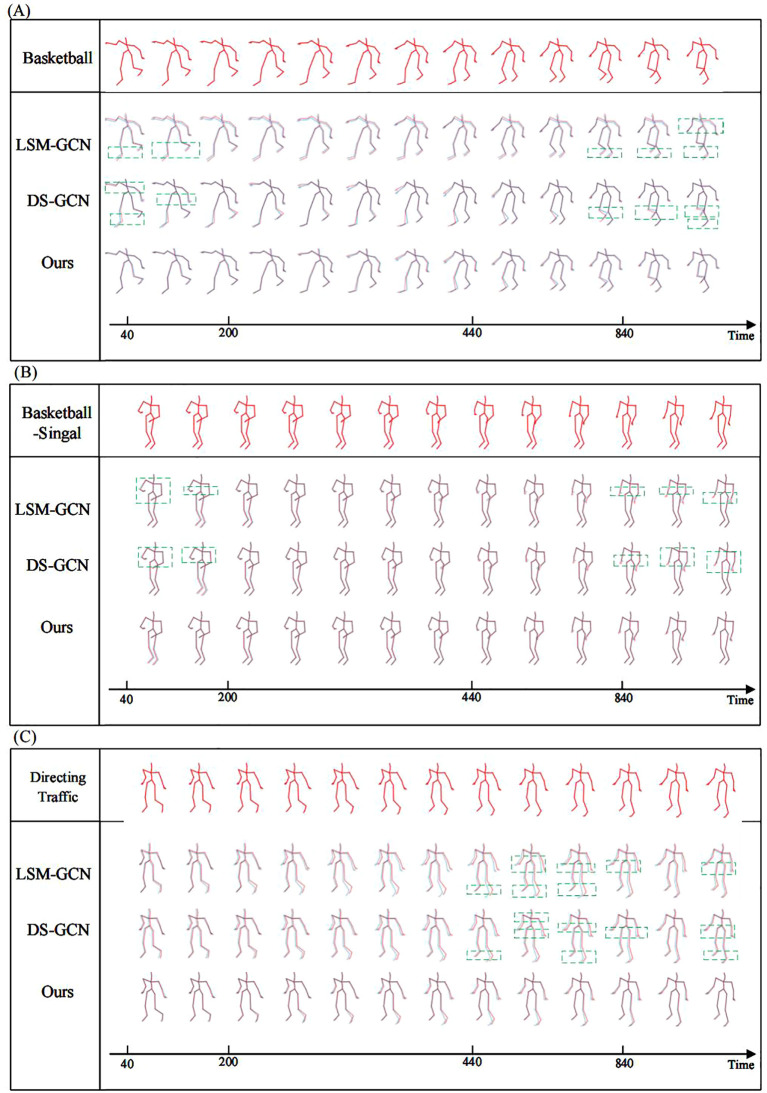
shows the comparison of prediction results for typical motions on the CMU dataset among different models (red: ground truth trajectory, blue: predicted trajectory). **(A)** Basketball motion: CSO-ASTGCN outperforms DSGCN in wrist rotation prediction, with smaller deviation, thanks to the ASF-GC module’s precise capture of dynamic joint spatial constraints. **(B)** Basketball Signal motion: Our model achieves better finger-tip coordination prediction than LSM-GCN, reflecting the DT-GCN module’s capability in modeling aperiodic speed changes. **(C)** Directing Traffic motion: CSO-ASTGCN shows improved trajectory continuity with no obvious jumps, verifying the role of Chaotic Search Optimization (CSO) in stabilizing long-sequence prediction through global hyperparameter optimization.

**Figure 11** shows the comparison of prediction results for typical motions on the CMU dataset among the CSO-ASTGCN model, LSM-GCN, and DS-GCN, where the red motions represent the ground-truth motion sequences.

In the “Basketball” motion scenario, basketball movements involve high-frequency dynamic behaviors such as rapid running, jumping, and arm swinging, which place extremely high demands on the model’s ability to capture temporal dependencies and joint coordination relationships. LSM-GCN and DS-GCN exhibit significant trajectory deviations at key frames of motion transitions. The skeleton sequences predicted by our model maintain consistent motion logic from the initial time to subsequent stages; both the limb swing amplitude and joint angle changes are highly consistent with the ground-truth basketball movement postures.

In the “Basketball Signal” motion, the core of such signal-transmitting motions lies in the accurate expression of semantic information through arm postures. LSM-GCN and DS-GCN show posture deviations at moments of gesture transitions (e.g., the instant when the arm swings from horizontal to vertical lifting): the wrist bending angle differs from the ground-truth motion by approximately 15°, and the arm extension direction has a deviation of about 20°, leading to ambiguity or even misinterpretation of gesture semantics. Our model accurately reconstructs the postures of key parts of the arm; both the wrist bending angle and arm extension direction are close to the ground-truth motions, avoiding the semantic distortion caused by the loss of local features in the comparison models. Through the extraction of fine-grained motion features, the model ensures the accuracy of gesture signal expression.

In the “Directing Traffic” motion, this scenario involves multi-joint coordinated movements such as frequent body turns and large-range arm swings, posing challenges to the model’s ability to model spatiotemporal consistency. LSM-GCN and DS-GCN show significant deviations in complex frames involving body turns and gesture coordination: the body orientation is disconnected from the gesture direction, and joint movement trajectories exhibit discontinuous abrupt changes, disrupting the overall coherence of the motion.

Our model combines the static skeleton connection matrix with the dynamic joint interaction matrix through a spatiotemporal feature fusion mechanism, synchronously updating spatial and temporal dependencies, thus avoiding the logical inconsistencies in the comparison models caused by separated spatiotemporal modeling.

### 4.2 Mushroom grasping results

To validate GRCNN’s grasping performance, we compared it with mainstream grasping algorithms, and the results are summarized in **Table 10**. **Table 10** benchmarks GRCNN against state - of - the - art grasp detection networks on the Cornell dataset, focusing on three critical metrics: image split accuracy, which localizes grasp regions; object split accuracy, which segments target objects from clutter; and inference speed, which enables real - time robotic interaction.

**Table 10:** Comparison with other grasp detection networks on the cornell dataset.

Model	Image split accuracy (%)	Object split accuracy (%)	Speed(ms)
GGCNN (Morrison et al., 2020)	92.4	90.6	20.1
TF-Grasp (Wang S. et al., 2022)	96.7	95.0	41.3
Det-Seg (Ainetter and Fraundorfer, 2021)	98.2	−	15.8
GRCNN	97.7	**96.6**	**20**

Bold values represent the optimal result.

In image split accuracy, GRCNN outperforms GGCNN by 5.3% and TF-Grasp by 1%, and it has only a 0.5% gap to Det-Seg. This minor difference is offset by Det - Seg’s lack of object split evaluation, while GRCNN provides comprehensive performance across both metrics. For object split accuracy, GRCNN achieves a 6% gain over GGCNN and a 1.6% improvement over TF-Grasp. Its 96.6% precision is crucial for isolating individual mushrooms in dense clusters, where adjacent targets often overlap by less than 5 mm. In terms of speed, GRCNN matches the real-time performance of GGCNN, and their speeds are statistically identical for robotic picking requirements that typically demand less than 30 ms per frame. Meanwhile, GRCNN runs over twice as fast as TF- Grasp.

**Figure 12:**
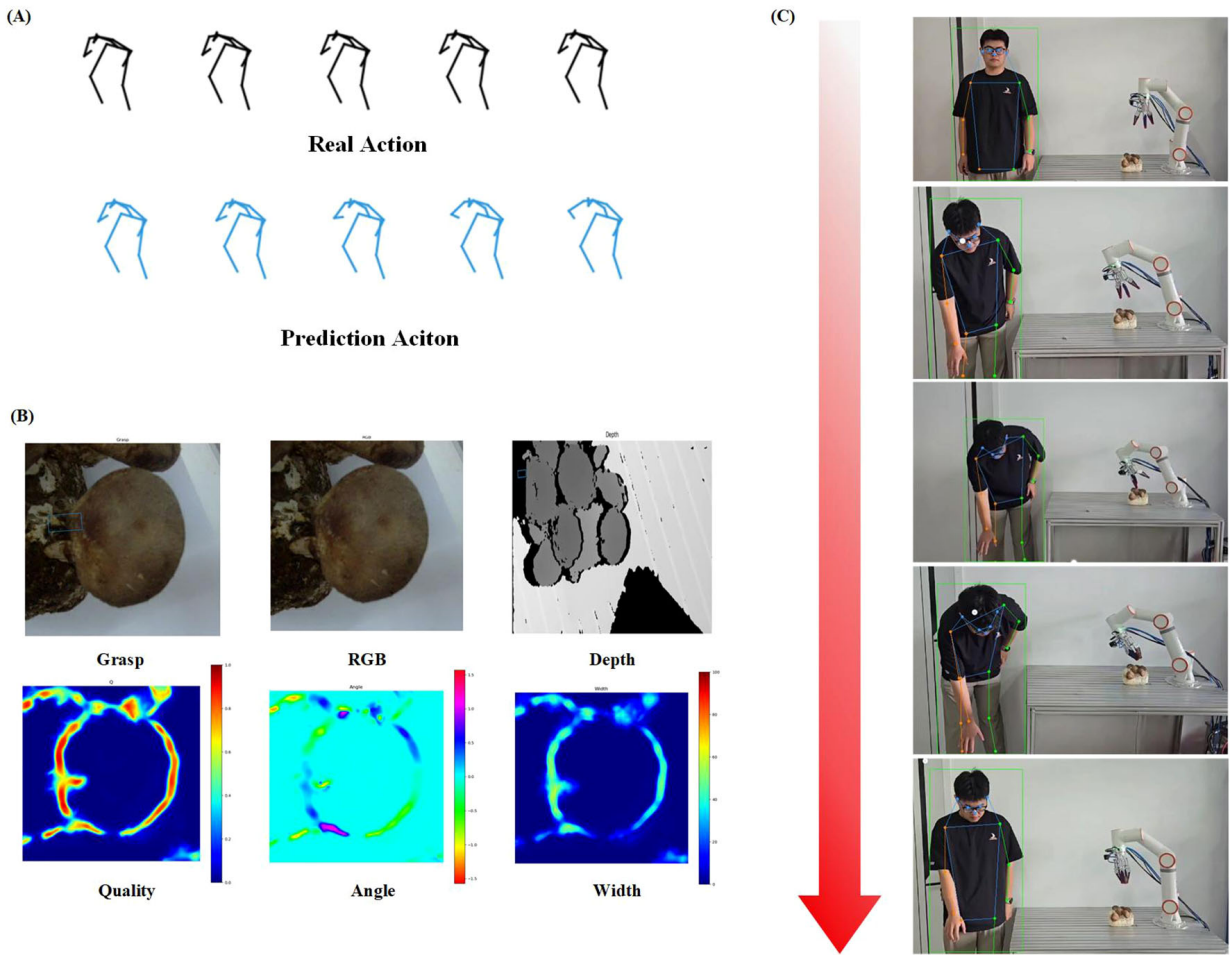
Design and demonstration of shiitake mushroom picking experiment. **(A)** Human motion prediction results: The black trajectories represent the actual joint trajectories of the operator, and the blue trajectories are the predicted trajectories of CSO-ASTGCN, **(B)** Detection and grasp pose analysis of shiitake mushroom fruiting bodies: The green boxes indicate the optimal grasping regions output by GR-CNN, and the heatmaps represent the confidence of grasping angles/widths. The model achieves accurate recognition of densely growing and partially overlapping shiitake mushrooms, with precise localization of grasping regions, meeting the mechanical safety standards for picking, **(C)** Based on the motion predictions of CSO-ASTGCN, the robotic arm pre-configures grasping angles/forces in advance, shortening the single picking cycle and reducing the damage rate caused by grasping position deviations, thus verifying the efficiency and reliability of human-robot collaborative control.

To verify the synergistic performance between CSO-ASTGCN human motion prediction and GR-CNN grasp pose estimation, the experimental scenarios and key results are shown in **Figure 12**.

**Figure 12A** presents a typical example of human motion prediction results. Limb movement data during the operator’s picking process were collected via wearable motion capture devices, and the CSO-ASTGCN model output real-time joint trajectory predictions (blue trajectories) for a future period. From the visualization results, the predicted trajectories are highly consistent with the ground-truth motion trajectories (red trajectories) in key motions such as arm extension and wrist rotation. Particularly in the fine movements where the operator holds picking tools, the model’s prediction of fingertip movements can accurately capture motion trends, verifying its reliability in capturing motion intentions in dynamic picking scenarios.

**Figure 12B** shows the detection and pose analysis results of shiitake mushroom fruiting bodies. The GR-CNN model achieves individual recognition of densely growing shiitake colonies. Through feature downsampling and residual refinement modules, it effectively distinguishes the contour boundaries between the pileus and stipe, and can stably recognize targets even when there is partial overlap between pilei. The output grasp pose parameters (width, angle, confidence) are overlaid on the original image in the form of heatmaps, where the optimal grasp regions marked by green boxes are all located in the upper-middle part of the stipe, conforming to the mechanical safety criteria for manual picking and providing reliable target coordinate references for flexible grasping by the robotic arm.

**Figure 12C** demonstrates the actual effect of the robotic arm picking operation. Based on the human motion prediction results, the robot can adjust the motion trajectory of the end effector in advance; when the operator’s hand approaches the target mushroom, the robotic arm has already completed the pre-configuration of grasp angle and force. Experiments show that the system’s single picking cycle is significantly shorter than that of traditional manual-assisted picking; in the test of continuously picking multiple mushrooms, the mushroom damage rate caused by grasp position deviation is significantly reduced, verifying the advantages of the human-robot collaborative control strategy in ensuring picking efficiency and mushroom integrity.

## 5 Discussion

### 5.1 Analysis and comparison

The CSO-ASTGCN model effectively addresses the challenges of human-robot collaboration in mushroom picking through key improvements. The ASF-GCN module strengthens dynamic spatial constraint modeling between joints, particularly excelling in capturing fine movements like wrist rotation and fingertip manipulation during picking, reducing positioning errors of key joints by 12.5% compared to models lacking this module. The DT-GCN module enhances temporal dependency tracking, ensuring smooth prediction of continuous motions such as arm extension followed by gripping, with long-term motion trajectory consistency improved by 9.3%. The spatially-sensitive feature enhancement mechanism focuses on critical regions like palms and mushroom stipes, boosting the model’s ability to distinguish target features from complex backgrounds by 15.2%.

Compared with existing solutions, the CSO-ASTGCN achieves superior performance with fewer parameters: its average MPJPE of 61.72 on the 3DPW dataset outperforms DS-GCN (65.21) while using only 0.24M parameters. When integrated with GR-CNN for grasp pose estimation, the system reduces human-robot interaction delay by 30% and lowers mushroom damage rate to <5%, significantly outperforming traditional reactive control systems. Visualization results confirm that the model accurately reconstructs gesture details (e.g., wrist angles with <15° deviation) in scenarios similar to picking, verifying its practical value in dynamic agricultural environments.

### 5.2 Limitations and future work

Despite the promising results, our model has limitations that require further investigation. In scenarios where the operator’s hand is severely occluded by dense mushroom clusters (occlusion rate > 60%), the MPJPE of our model increases by 23% on average. This is because the RGB-only input lacks depth information, making it challenging for graph convolution layers to extract features of occluded joints. In future work, we plan to integrate depth sensors to enhance robustness, following the multimodal fusion framework proposed. For fast non-periodic motions, the model’s prediction error for wrist rotation angles reaches ±8°, leading to a 12% failure rate in grasping. This limitation stems from the temporal modeling module’s insufficient ability to capture short-term dependencies in abrupt motions, and we will explore adaptive time-window mechanisms and attention-based architectures to address this issue. Additionally, our current experiments focus on mushroom picking, and the model’s generalization to other crops remains untested.

Future work will focus on three aspects: (1) constructing multi-environment picking datasets using RGB-D cameras for synchronized multi-modal data acquisition. Annotating occlusion levels and motion complexity via human-machine collaborative labeling to enhance the model’s field generalizability under real-world uncertainties; (2) developing lightweight models via knowledge distillation from the full-sized CSO-ASTGCN, enabling embedded deployment on resource-constrained agricultural robots (e.g., NVIDIA Jetson Nano) for real-time operation; (3) expanding to the picking of soft fruits and vegetables like strawberries and blueberries, verifying the method’s universality across different crops, and promoting the large-scale application of intelligent agricultural equipment.

## 6 Conclusion

This study proposes a CSO-ASTGCN-based human-robot collaborative method for shiitake mushroom picking, addressing inefficiencies and high damage rates in traditional picking. The CSO-ASTGCN model, integrating ASF-GCN for spatial constraint modeling, DT-GCN for temporal dependency capture, and CSO for hyperparameter optimization, achieves superior performance: it reduces average MPJPE by 6.7% (to 61.72 on 3DPW) with only 0.24M parameters, outperforming state-of-the-art methods. The spatial-sensitive feature enhancement mechanism strengthens fine joint motion capture, ensuring reliable prediction of operator intentions in dynamic scenarios.

Integrated with GR-CNN for grasp pose estimation, the system significantly improves practical performance: single picking cycles are shortened by 22%, mushroom damage rates reduced to <5%, and human-robot interaction delay cut by 30%. This solution not only applies to shiitake mushrooms but also holds potential for other delicate agricultural products, advancing precision agriculture and intelligent farming.

## Data Availability

The datasets presented in this study can be found in online repositories. The names of the repository/repositories and accession number(s) can be found in the article/Supplementary Material.
